# Topographic stress controls fissure segmentation and eruptive cone formation during the Laki and Eldgjá eruptions

**DOI:** 10.1038/s43247-026-03980-4

**Published:** 2026-09-02

**Authors:** Maria Hurley, Francesco Maccaferri, Thomas R. Walter

**Affiliations:** 1 GFZ Helmholtz Centre for Geosciences, Potsdam, Germany; 2 Institute of Geosciences, University of Potsdam, Potsdam, Germany; 3 Istituto Nazionale di Geofisica e Vulcanologia (INGV), Sezione di Bologna, Bologna, Italy

**Keywords:** Natural hazards, Volcanology, Tectonics

## Abstract

Elongated dykes in tectonic rifts feed extensive basaltic fissure eruptions, yet surface eruption sites are highly discontinuous and unpredictable. Here we show that along-strike topographic variations, and the associated stress gradients, correlate with this localisation. We investigate eruptive cones at the two most productive historical basaltic eruptions in Iceland, Laki (1783–1784) and Eldgjá (~939–940). Morphological analyses of  > 300 cones reveal volume variations (10^0^–10^7^ m^3^) and segmentation along  ~ 100 km of fissures. Comparing cone distribution and volumes along the rift zone with pre-eruptive stress models, we demonstrate that high topographic stress gradients inhibit cone growth. We identify a critical stress gradient threshold predicting where larger cones are inhibited, while horizontal stress gradient peaks predict the location of fissure gaps. Our findings establish an empirical and physical link between topographic stress and volcanic morphologies, complementing previous models with a quantitative framework that aims at improving the forecasting of along-rift vent locations and development.

## Abstract (web summary)

Variations in eruptive cone volume and fissure-gap locations are associated with topographic stress gradients, providing quantitative support for a link between topographic stress and volcanic morphology, according to morphometric and statistical analysis of eruptive fissures in Iceland.

## Introduction

Basaltic fissure eruptions are usually supplied by underlying dykes, indicating brittle fracture zones in the crust, which may propagate laterally over tens of kilometres^1,2^. Notably, even when a single lateral dyke represents the feeding system for a rift eruption^3,4^, the rift-related fissures forming at the surface are commonly expressed as highly segmented and spatially discontinuous, with eruptive vents and surface rupture remaining localised to discrete sections of the overall structure. This behaviour has been documented for the 2005 Dabbahu rifting episode in the Afar Depression, where a ~60-km-long dyke intrusion produced deformation and eruptive activity concentrated within a limited segment^4^. Similar patterns of localised vent formation along otherwise continuous fissure systems are observed in Icelandic rift zones, such as the 1975–1984 Krafla^5^ and 2014–2015 Bárðarbunga–Holuhraun^3^ events, and more recently in the 2021–2026 eruptions on the Reykjanes Peninsula^6,7^. Similar segmentation occurs in other tectonic settings, including the oceanic shield volcanoes of Kilauea in Hawaii^8^ and Piton de la Fournaise in La Réunion^9^. These observations indicate that along-strike variations in stress conditions, magma supply, and crustal structure commonly partition deformation and eruption into discrete segments along longer rift structures.

In divergent settings, regional tectonic stress typically dictates the primary trend of these intrusions, which is commonly perpendicular to the spreading direction, corresponding to the axis of least compressive stress^10^. Superimposed on this regional trend, surface topography affects magma dynamics by locally altering the stress orientation and magnitude through loading and unloading mechanisms^11,12^. It has been proven that large volcanic edifices induce compressive stresses that can divert or arrest upward propagating dykes^3,13–15^, while mass removal (unloading by erosion or collapse) can create stress traps that lock vertical dyke propagation and promote horizontal sill formation^16,17^. This deterministic relationship makes the study of crustal stress critical for predicting dyke trajectories, potential magma-stalling locations, and ultimately the position of future vents^15,17,18^. For the along-rift intrusions studied in this work, the tectonic extensional stress remains the dominant control, as the intrusions are vertical and rift-parallel, following the stress regime established by the rift spreading. However, shallow topography-induced stress heterogeneities may still influence the confining stress gradient acting on the upper part of the vertical dyke walls, affecting the magma overpressure gradient and flow dynamics^14^, possibly focusing magma flow toward specific surface locations.

As a result, the geometry and spatial arrangement of volcanic landforms serve as a permanent record of subsurface dyke dynamics. At basaltic fissure eruptions, relatively small, steep-sided volcanic landforms develop, mainly scoria cones and spatter cones^19,20^. Scoria cones form from the accumulation of loose, cooler pyroclasts during Strombolian activity, while spatter cones develop during Hawaiian-style fountaining through the rapid accumulation and agglutination of hot and fluid lava clots^20,21^. These landforms are often asymmetric, driven by factors such as strong wind directions during deposition, slopes and eruption dynamics^19,21,22^. Furthermore, their geometry can provide a high-resolution proxy for eruptive energy and magma stalling locations^23^. For simplicity, we refer to all these monogenetic features herein as eruptive cones. This surface–subsurface link is further supported by observations of active eruptions, where surface deformation patterns from geodetic data (e.g., GNSS, InSAR) correlate directly with the volume and geometry of the feeding intrusion^4,24^.

**Fig. 1:**
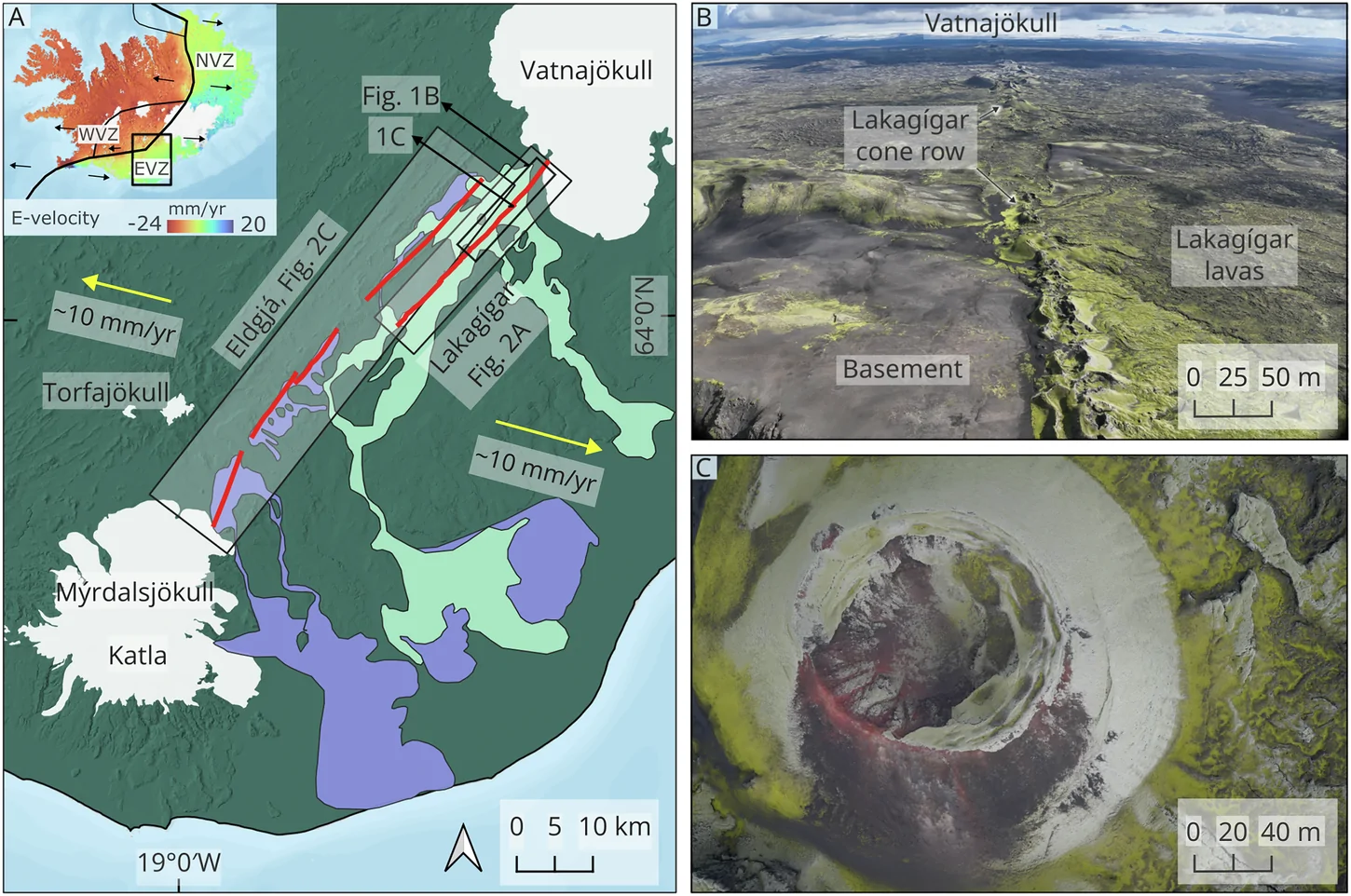
Setting of the study area, the largest historic fissure eruptions in Iceland. **A** Map of the Eldgjá and Lakagígar fissures in the East Volcanic Zone. The lavas from the Eldgjá and Laki eruptions are indicated in green and purple, respectively (after refs. ^40, 30^). Yellow arrows indicate the total plate divergence rate of ~20 mm/year^84^. Inset: Sentinel-1 InSAR near-east velocity map from plate spreading and glacial isostatic adjustment^85^. WVZ, NVZ and EVZ: West, North, and East Volcanic Zones. **B** Field photo of NE Lakagígar eruptive cone alignment, showing contact between basement and lava flows. **C** Morphology close-up of a Lakagígar scoria cone.

Despite extensive research on dyke propagation and fissure dynamics in Hawaii^8^, the East African Rift^4,14^, La Réunion^25^, the Canary Islands^26^ and Iceland^3,27,28^, a quantitative framework explaining how topography may interact with long and discontinuous eruptive fissures remains overlooked. This study investigates magma–topography interactions by analysing two of the Earth’s most volumetrically productive historical basaltic events: the Eldgjá (~939–940^29^) and Laki (1783–1784^30^) eruptions. Located in Iceland’s East Volcanic Zone (EVZ; Fig. 1), these eruptive fissures were fed by the lateral flow of basaltic magmas from high-level reservoirs located beneath central volcanoes: Katla for Eldgjá, located under Mýrdalsjökull (Fig. 1A), and Grímsvötn for Laki, located under the Vatnajökull ice cap (Fig. 1A)^31^. The fissures interacted significantly with pre-eruptive topographic relief over tens of kilometres, providing an ideal setting to isolate the effects of surface loading. Although <10 km apart, subparallel (~NE–SW), and sharing an identical tectonic setting, they exhibit contrasting fissure architectures. This offers a unique opportunity to decouple regional stress from topographic controls. We integrate high-resolution satellite and drone data with analytical stress models to evaluate topographic controls on fissure segmentation and quantify the interplay between topographic loading and the spatial distribution and morphology of eruptive centres. Ultimately, we propose a quantitative framework for fissure eruptions based on topographic stress to evaluate zones susceptible to large cone growth versus areas where eruptions will be mechanically inhibited.

## Results

The Laki (1783–1784) and Eldgjá (~939–940) eruptions represent the most voluminous basaltic events in the Common Era. Fed by the central volcanoes of Grímsvötn in the east (Laki) and Katla in the west (Eldgjá), they produced ~14.7 and ~19.7 km^3^ of lava, along with >0.4 and >0.3 km^3^ of tephra, respectively^30,32^. After the start of each eruption, the opening of the feeder dykes of both eruptions migrated northeastward, evolving from initial explosive and phreatomagmatic activity to magmatic phases characterised by Strombolian, sub-Plinian and Hawaiian fountaining^30^.

The fissures, Lakagígar (~27 km) and Eldgjá (~70 km), consist primarily of eruptive cone rows (scoria and spatter cones) bounded by volcano-tectonic grabens driven by dyke emplacement and spreading, commonly covered by lavas^30,33,34^. However, the architecture and eruptive cone morphology along the fissures differ. Lakagígar is largely continuous, well-preserved and with only ~100–300 m gaps within Mount Laki. Its eruptive cones are typically equilateral and comparatively small (diameters from a few metres to ~1 km). In contrast, Eldgjá exhibits a more complex architecture characterised by kilometre-scale gaps and en-échelon fissure jumps (here referred to as segmentations for simplicity). Eldgjá eruptive cones are generally larger (up to ~2 km diameter) and morphologically complex, often featuring craters elongated along strike.

### Spatial distribution and segmentation of eruptive cones

**Fig. 2:**
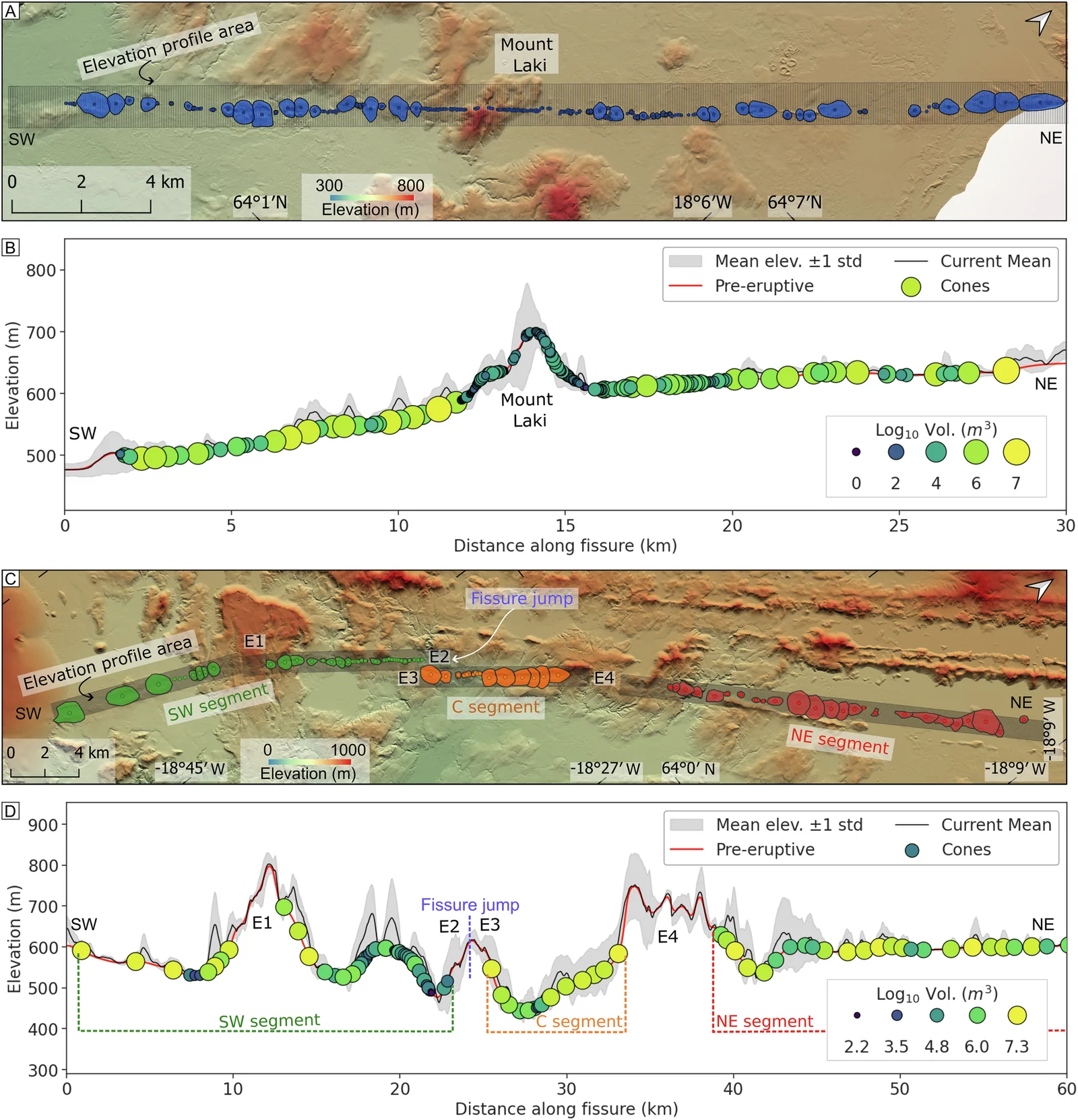
Topography and cone volume distribution of eruptive cones along the Lakagígar and Eldgjá eruptive fissures. **A** Hillshade digital elevation model (DEM) of the Lakagígar area. Blue polygons indicate the 226 delineated cones. The grey rectangular polygon represents the 1200-m-wide swath used to generate the elevation profile in (**B**). **B** SW–NE along-fissure elevation profile of Lakagígar. Overlaid markers represent the mapped cones' centroids, size and colour coded according to the logarithmic scale of the calculated volume. **C** Hillshade DEM of Eldgjá and delineated 85 cones. The eruptive fissure is divided into three descriptive segments: SW (green), C (orange) and NE (red). Prominent topographic highs linked to fissure segmentation are labelled E1–E4. The swath area considered for the elevation profile is also marked in grey. **D** SW–NE along-fissure elevation profile of Eldgjá. Similar to (**B**), projected cones are overlaid and coded according to the log-scaled volumes.

We analysed the spatial distribution of an inventory of 226 and 85 eruptive cones along the Lakagígar and Eldgjá fissures, respectively (Fig. 2). At Lakagígar, cone volumes range from ~1 to 10^7^ m^3^ and show an inverse correlation with elevation (Fig. 2A, B). The fissure segment and cone alignments located to the southwest of Mount Laki lie at the lowest elevations and host the most voluminous cones (>10^5^ m^3^) (Fig. 2B). In contrast, the central sector, located around Mount Laki, corresponds to the maximum pre-eruptive elevation and exhibits a marked reduction in eruptive cone volume, characterised by clusters of small cones (<10^2^ m^3^) and minor discontinuities (Figs. 2B and S2 in the Supplementary information). Finally, the northeastern extension represents an intermediate scenario, where moderate elevations host cones of intermediate to large volumes (Fig. 2B).

At Eldgjá, where eruptive cone volumes range from 10^2^ to >10^7^ m^3^, the architecture is more complex and divided into three distinct segments (Fig. 2C). The southwest (SW) segment, near Mýrdalsjökull, consists of spaced, large vents (>10^6^ m^3^). This segment is interrupted by topographic peak E1, where a 4-km gap develops. Beyond the gap, the fissure reappears with smaller, more densely distributed vents before terminating at peak E2. Here, an en-échelon jump marks the transition to the central (C) segment. Bounded by topographic peaks E2 and E3, this section displays a heterogeneous cone volume distribution but is dominated by a deep topographic depression. Finally, the northeast (NE) segment features a fragmented architecture with generally smaller, poorly preserved cones. Unlike Lakagígar, where elevation seems to modulate cone size, topography at Eldgjá primarily causes segmentation (Fig. 2C, D). The fissure trace is systematically interrupted by major topographic peaks (E1–E4), where the highest elevations and steepest slopes result in spatial gaps. In contrast, over areas of moderate gradients in elevation, such as between E1 and E2, the fissure remains continuous, but activity is restricted to clusters of small-volume cones only, similar to the pattern observed at Mount Laki.

### Topographic stress models

**Fig. 3:**
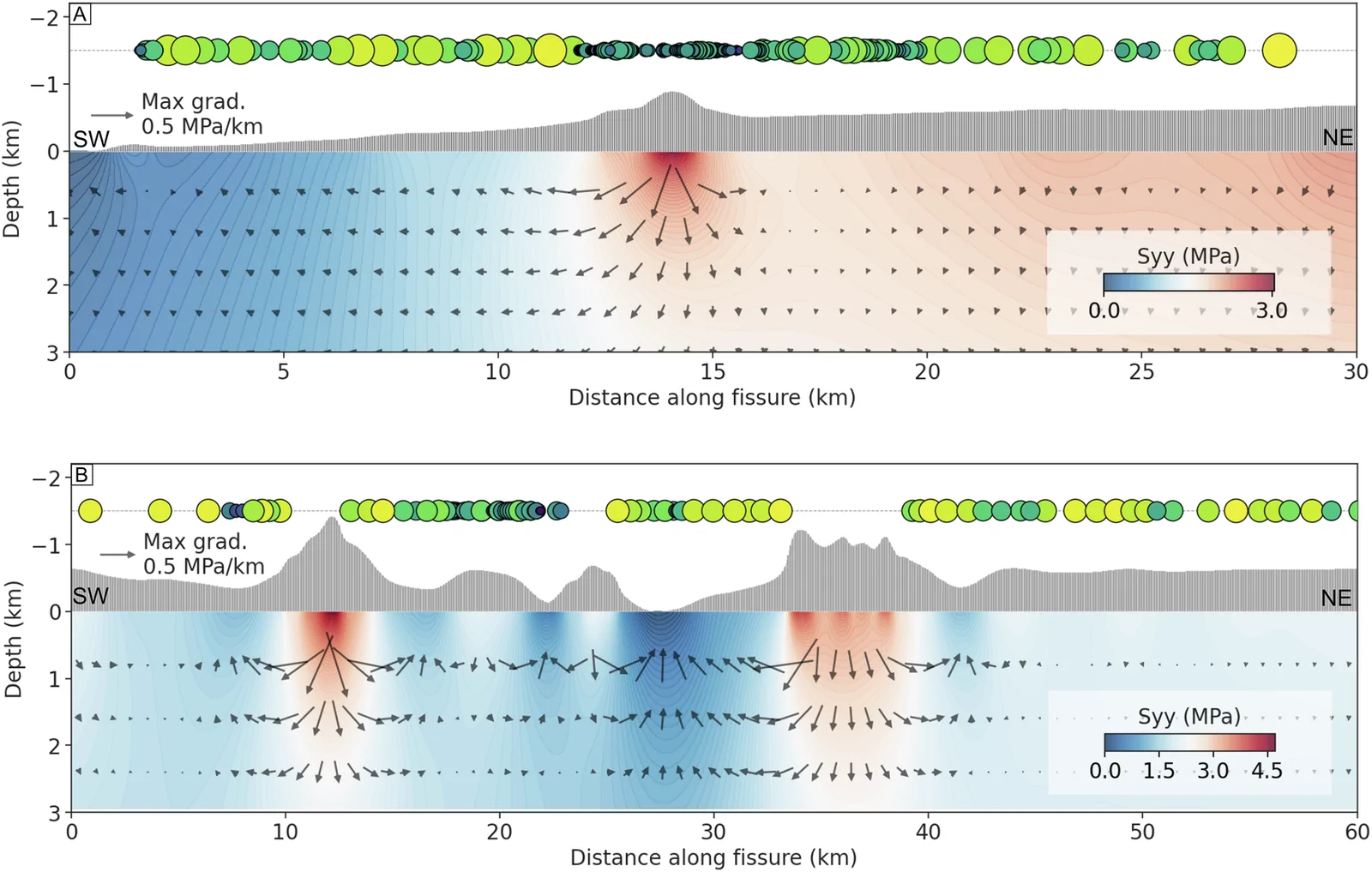
Topographic out-of-plane stress (*S*_*yy*_) models and cone volume distributions for the Lakagígar and Eldgjá fissures. Stress field models for **A** Lakagígar and **B** Eldgjá after ref. ^74^ apply the reconstructed pre-eruptive topography as 50-m-wide vertical strip loads to an elastic half-space (shown in grey, see the section “Topographic stress models” for more details). Vertical scale is exaggerated 4× for visualisation purposes. The models indicate stress magnitudes with colour contours; red indicates high compression. Black arrows show maximum stress gradient vectors, indicating favoured magma migration paths toward lower stress regions. For comparison purposes, we plotted the along-fissure cones above the models, size- and colour-coded consistently with Fig. 2. Colour scales are 0–3 MPa for Lakagígar (**A**) and 0–4.5 MPa for Eldgjá (**B**), reflecting the greater stress magnitudes and gradients in the latter.

To investigate the drivers of the fissure architecture, we analysed the stress field induced by topographic loading of the crust along both fissures using the reconstructed along-strike pre-eruptive elevation profiles (see the subsection “Pre-eruptive topography reconstruction”). We focused on the stress component perpendicular to the dyke plane (*S*_*yy*_), which affects the magma overpressure gradient over the dyke plane, and compared this with the cone volume distribution and segmentation (Fig. 3).

The stress models along both fissures revealed two main trends. First, smaller-volume cones tend to cluster at local topographic highs, where large stress magnitudes and gradients occur close to the surface (Fig. 3). This trend is particularly evident at Mount Laki in the Lakagígar fissure (Fig. 3A) and at the topographic high located between ~15 and 23 km distance along the fissure, between E1 and E2 in Eldgjá (Fig. 3B). In contrast, cone volumes are more variable in areas of relatively flat topography, where topography-induced stresses are lower.

The second key trend relates to the eruption gaps observed by a lack of cones at Eldgjá (Fig. 3B). Here, the steep slopes bounding topographic highs (ranging between 6° and 19° at the onset of the gaps) induced sharp horizontal changes in the stress gradient at depth, locally reaching 2 MPa/km on the SW slope of E4. These stress gradient peaks, associated with high topographic loading and stress magnitudes, spatially coincided with the segmentation sites, i.e., locations where the eruptive fissure is interrupted by a spatial shift, sometimes accompanied by a change in orientation. In contrast, the gentler slopes at topographic highs in Lakagígar (averaging 0.7° and reaching a maximum of 7° along the profile, Fig. 3A) resulted in significantly lower gradients that remain below 1 MPa/km.

### Quantitative comparison with cone volumes and fissure segmentation

#### Lakagígar: Cone volume distribution

**Fig. 4:**
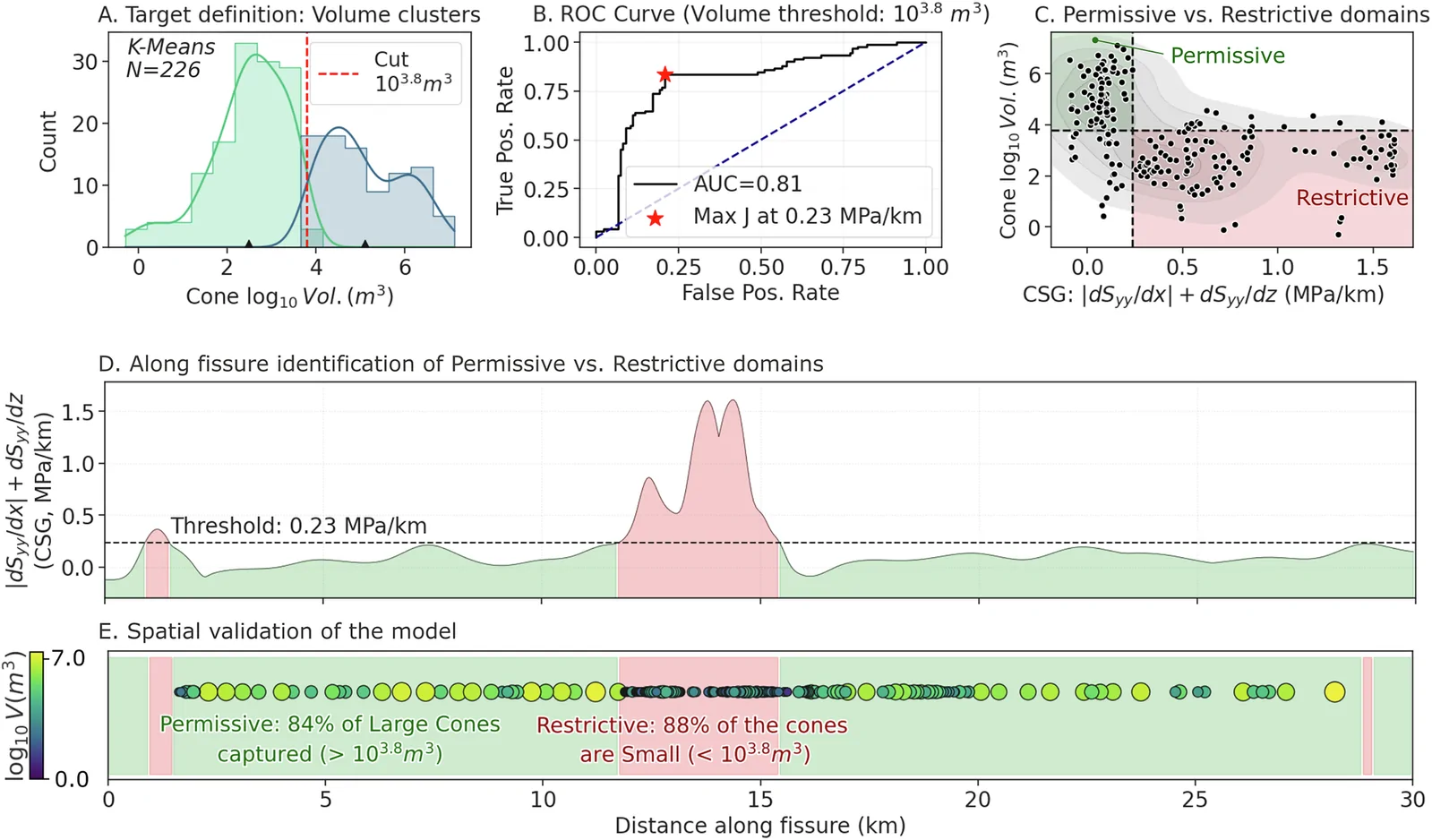
Quantified topographic stress controls on eruptive cone volumes at Lakagígar. **A** Histogram of cone volumes (*N* = 226, where *N* represents the total number of individual eruptive cones mapped along the Lakagígar fissure). The vertical dashed line indicates the volume cutoff 3.8 (m^3^, log scale); black triangles indicate the centroid of each cluster, defined by *K*-means. **B** ROC curve analysis identifying the optimal CSG threshold at 0.23 MPa/km. **C** Scatter plot of log-cone volume vs. CSG. The threshold divides the field into Permissive (green) and Restrictive (red) domains, favouring and inhibiting cone volume growth, respectively. **D** Along-strike profile of the CSG, obtained by averaging the top 0.5 km of the stress models. After applying the CSG threshold, Permissive and Restrictive regions are spatially defined along the fissure. **E** Spatial validation of the model. The map compares predicted domains with observed cone volumes; 84% of large cones are captured in the Permissive domain, while 88% of cones in the Restrictive domain are small.

To translate these observations into a classification framework, we developed a binary classification system to identify domains that inhibit or favour the formation of the larger volume cones. We found that a Combined Stress Gradient metric CSG $=\left\vert {\rm {d}}{S}_{yy}/{\rm {d}}x\right\vert +{\rm {d}}{S}_{yy}/{\rm {d}}z$, which sums the horizontal and vertical confining-stress gradients, provided the strongest predictive power for the location of ’large’ vs. ’small’ cone populations among all tested metrics (see Figs. S3, S4 in the Supplementary information). To capture the influence of shallow crustal stress on magma transport, we calculated the CSG parameter by averaging the stress values within the uppermost 0.5 km. Our sensitivity analysis confirmed that results remain stable considering depth ranges between 0–0.2 and 0–3.0 km (Figs. S3 and S4). This demonstrates that using a depth-averaged metric from the surface to the target depth, like the CSG, preserves the shallow topographic signal across the target depths we tested. We first tested the model using the Lakagígar cones (*N* = 226, Fig. 4). We used a *K*-means clustering algorithm to define a volume cutoff separating the cones population into ’large-’ and ’small-volume’ clusters (Fig. 4A). The volume cutoff was identified at ~6.3 × 10^3^ m^3^ (10^3.8^ m^3^). With this volume cutoff, we performed a receiver operating characteristic (ROC) analysis to determine the optimal CSG threshold to segregate the clusters (AUC = 0.81; Fig. 4B). With this approach, we explored all possible CSG thresholds to find the best balance between true positives (large cones below the threshold) while keeping false positives (small cones below the threshold) to a minimum. The resulting optimal threshold is CSG = 0.23 MPa/km. We tested the effectiveness of the results, yielding a Youden’s *J* Index of 0.63.

Applying this CSG threshold spatially defined two crustal domains, referred to as a Permissive domain (below threshold), where a low CSG favoured the development of large cones, and Restrictive domain (above threshold), where high CSG inhibited large cone growth, limiting activity to small cones (Fig. 4C). When applying this threshold along the fissure, 84% of large cones fell correctly within Permissive domains, while 88% of the cones located in Restrictive domains were small (Fig. 4D, E).

#### Eldgjá: cone volume distribution and fissure-gap prediction

**Fig. 5:**
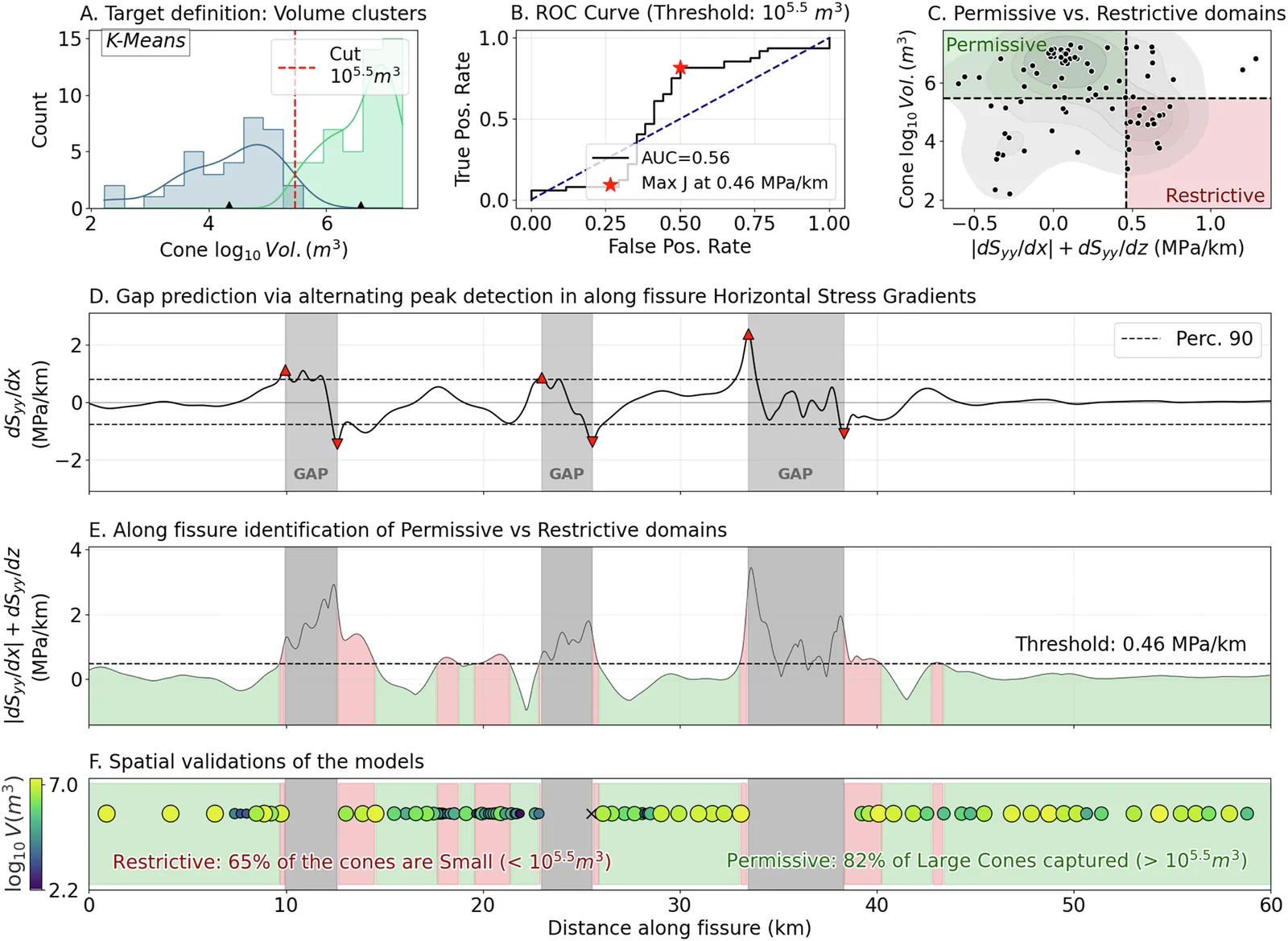
Quantified topographic stress controls on cone volume and segmentation at the Eldgjá fissure. **A** Histogram of cone volumes (*N* = 85, where *N* represents the total number of individual eruptive cones mapped along the Eldgjá fissure). The vertical dashed line indicates a volume cutoff of 5.5 (m^3^, log scale), defined by *K*-means. **B** ROC curve analysis identifying the optimal CSG threshold at 0.46 MPa/km. **C** Scatter plot of log-cone volume vs. CSG. The threshold divides the field into Permissive and Restrictive domains. **D** Along-strike profile of the horizontal stress gradient. Alternating maximum and minimum values, resulting from steep slopes, are detected for gap prediction; dashed lines mark the 90th percentile threshold used for peak detection. **E** Based on the CSG threshold, Permissive and Restrictive domains are spatially defined along the fissure. **F** Spatial validation of the model: Permissive domains capture 82% of large cones, 65% of small cones fall within Restrictive domains. The gaps are accurately predicted, with one single cone located within a predicted gap (marked with a black cross).

We applied this stress-gradient analysis approach and the classification framework to the Eldgjá fissure cones (*N* = 85), a much older separate system with larger morphological and structural complexity. First, we defined the local volume cutoff at ~3 × 10^5^ m^3^ (10^5.5^ m^3^), using *K*-means clustering (Fig. 5A). ROC analysis showed a lower performance compared to Lakagígar, with an AUC of 0.56 and a maximum Youden’s *J* Index of 0.32 at a CSG threshold of 0.46 MPa/km (Fig. 5B). Applying this threshold separated the cone population into distinct stress domains (Fig. 5C). Mapping the CSG threshold spatially along the fissure CSG profile distinguished between Restrictive and Permissive domains (Fig. 5E). Comparing these mapped domains with the observed cone distribution showed that 82% of the large cones clustered within the Permissive domain, while 65% of the cones found in the Restrictive domain were small (Fig. 5F). To account for sections lacking cone formation, we also tested a continuous spatial binning approach along the Eldgjá fissure (see Supplementary information for details). By stacking cone volumes over a sliding window along the fissure, we incorporated non-eruptive gaps as zero-volume data points, which indeed raised the model’s predictive performance (AUC = 0.65–0.72; Figs. S9–S11). Furthermore, we extended the model to predict fissure gaps. Analysis of the along-fissure horizontal stress gradient reveals sharp, alternating peaks corresponding to steep, opposing mountain slopes (Fig. 5D). Physically, the horizontal stress gradient represents the spatial rate of change in resistance encountered by the laterally propagating dyke. A sharp peak in this gradient, hence, represents a sudden increase in such resistance, and magma overpressure may suddenly become insufficient to maintain vertical fracture propagation and feeding of eruptions. This suppression in magmatic ascent would explain the initiation of an eruptive gap at the surface, and magma may continue propagating laterally at depth until a permissive stress environment is reached again. By applying a 90th percentile threshold to this gradient, we identified these peaks as lateral stress barriers, defining the predicted gaps between them. The gap prediction algorithm showed 99% accuracy, with only one single mapped cone falling within a predicted gap (marked with a cross in Fig. 5F).

## Discussion

The morphology and spatial distribution of eruptive vents along the ~100 km-long Lakagígar and Eldgjá fissures were analysed using high-resolution digital elevation models and slope maps, allowing the construction of a database of 311 eruptive cones and their associated pre-eruptive topography settings. While the drone-derived DEM (~20 cm/px) was useful to identify cones with volumes as small as 1 m^3^ in targeted areas, the regional ÍslandsDEM (2 m/px) imposes a higher minimum detection limit. However, such smaller structures are expected to be minimal and should not significantly change our results. Automated deep-learning detection tools, such as NETVOLC^35^, were initially tested to identify eruptive landforms. However, we found that the performance of these algorithms was highly sensitive to input parameters, particularly the window size, and to the morphology of the eruptive fissures themselves (e.g., cone coalescence). In practice, the models performed reliably only where cones were isolated and morphologically well-defined (Fig. S1 in the Supplementary information). To minimise these biases, we performed a comprehensive manual mapping of eruptive landforms. Although time-intensive, this workflow proved more suitable for resolving the complex and overlapping cone architectures characteristic of these large fissure systems. The resulting dataset allowed us to accurately identify and analyse the locations and dimensions of erupting cones and to reconstruct their relationships with the surrounding topography. We found that, in order for the automated tools to have worked reliably, an extensive training phase would have been needed, tuning input parameters to specific landform morphologies. Such an effort would likely become worthwhile only when analysing numerous fissures with comparable geomorphologic characteristics.

In order to link the surface morphology of the eruptions with subsurface mechanisms related to topography, we created topographic stress models. These rely on 2D analytical solutions for an elastic half-space, which represents a simplification of the three-dimensional nature of an active magmatic rift topography. Under the plane-strain assumption, deformation in the out-of-plane direction (parallel to the spreading direction) is suppressed, as the topography profile extends infinitely in the out-of-plane direction, leading to an overestimation in the absolute stress values^36^, and particularly the confining stress *S*_*yy*_ on the dyke plane. Nevertheless, the framework that we present in this work does not rely on absolute stress values, but rather on *S*_*yy*_ gradients, which influence the magma overpressure gradient on the dyke plane. In particular, the absolute stress values resulting from a 3D stress model are expected to decay faster with distance compared with a plane strain calculation (Fig. 11 in ref. ^36^), and therefore our stress gradients may represent a conservative estimate with respect to a full 3D calculation (a faster stress decay would result in an even steeper stress gradient). Additionally, our analysis focuses on the shallowest 0.5 km of the crust, and the topographic highs we measured (e.g., Mount Laki) have out-of-plane dimensions that at least double this shallow depth; the infinite out-of-plane assumption may represent a rather robust approximation for our case studies. Also, the 2D elevation transects used as input in our models correspond to the average elevations measured across 1200-m-wide swaths, thereby reducing the bias associated with linear cross-sections^37^.

Another assumption of our analytical models is the use of an elastic half-space, which represents the crust as homogeneous, ignoring mechanical layering. The upper crust in Iceland, and particularly in this region, is known to be composed of contrasting lithologies, typically alternating basaltic lava flows and hyaloclastites, with elastic properties that may vary several factors along the vertical^38^. While vertical layering deviates stress trajectories and may promote vertical dyke arrest or deflection^27^, we expect that incorporating such a layering into our models would not change our main results. Here we are analysing confining stress gradients to explain lateral, along strike variations such as fissure segmentation and cone volume distribution. These lateral variations in the confining stress gradients would not be significantly affected by the vertical stratification or layering.

A further methodological challenge lies in reconstructing the pre-eruptive topography. We used a filtering procedure that removed positive elevation anomalies. This approach efficiently removed young eruptive cones but did not perform with the same efficiency on broad accumulations of lava flows. However, the shallow stress gradients generated by surface loads are dominated by steep slopes and sharp topographic contrasts; the effect of smooth and relatively thin (<25 m on average^30,39^) lava flows is negligible.

Finally, our models are static and do not account for the temporal evolution of the eruptions. At both Lakagígar and Eldgjá, cone formation was sequential following discrete eruptive episodes generally propagating from southwest to northeast^30,40^. This implies that the stress field varies with time as dykes are emplaced and cones grow, dynamically affecting subsequent magma focusing and segmentation. Furthermore, as magma propagates laterally away from the source, processes like crystallisation and heat loss alter its rheology (e.g., increasing its viscosity). This means that applying a uniform stress threshold for defining Permissive and Restrictive domains along the entire fissure is a limitation. Despite these simplifications, the emergence of consistent patterns across both fissure systems and across multiple spatial scales suggests that the mechanical controls identified are dominant.

At a broad scale, the orientation, long-term evolution, and deep structural architecture of both the Lakagígar and Eldgjá feeding systems are fundamentally governed by the regional tectonic extension and remote stress fields. At crustal levels, the propagation paths of dykes are dictated by a combination of remote forces, including regional tectonic strain, strain accumulation from previous intrusions, and discontinuities^27,41,42^. Whether these feeding systems behave primarily as active, magma-driven fractures governed by internal magma overpressure or as passive following the regional extension remains unknown^8^. The deep-seated magmatic systems are modified by spatial or temporal heterogeneities in the remote stress field, though these regional-scale variations remain hard to constrain explicitly. Consequently, within our elastic modelling approach, remote stress forces were approximated as uniform to isolate the specific contribution of shallow surface loading. Petrological and historical evidence suggested that both eruptions were supplied by major, single-pulse laterally propagating dykes^3,30,40^. As magma moved laterally away from the central volcanic plumbing system, the effects of topographic stress gradients became superimposed on the regional stresses, altering the magma overpressure gradients within the dyke plane and therefore the magma flow near the surface. This local-scale superposition may be used to explain key features in the architecture, morphology, and evolution of the eruptive fissures, through two primary controls: (1) fissure gaps and segmentation and (2) cone volume spatial clustering. In summary, while the long-term crustal state of the rift is continuously modified by remote tectonic forces, our results demonstrate that shallow topographic-induced stress gradients may exert the final, localised filter on magma transport moments before breaching the surface.

By quantifying this shallow topographic effect, our results provide, to the best of our knowledge, a quantitative framework linking pre-eruptive surface topography to the morphology of fissure eruptions. The binary classification of the crust into Permissive and Restrictive domains based on the sum of vertical and horizontal confining stress gradients suggested that topographic loading controlled cone sizes along both fissures.

The receiver operating characteristic (ROC) analysis of cone size classification yielded a lower performance at Eldgjá than at Lakagígar (AUC = 0.56). This discrepancy arises because the reconstructed steeper morphology of Eldgjá generated stress gradients sufficiently large to completely arrest dyke propagation in some sectors. Therefore, high-stress restrictive regions in Eldgjá completely suppress eruptions (forming gaps) and are thus not captured in the classification performance metric. We showed that including fissure gaps in our analysis as zero-volume cones indeed improved the model’s performance (Figs. S9–S11).

Physically, the two domains represent the contrasting conditions governing magma transport within the propagating dyke. In Permissive domains, characterised by low CSG values, magma encounters limited resistance to vertical flow. In contrast, in Restrictive domains, higher CSG opposes upward propagation and may deflect magma laterally along the fissure. Magma flow along a fissure will focus where the heat supplied by the extruded magma is higher than the heat lost to the country rock^43^. Such a process, defined as thermal erosion^43^, is dictated by the width of the dyke. Previous studies^8^ suggest that cone spatial clustering is driven by fluid mechanics and thermal effects. Small variations in the initial aperture of the fissure amplify over time because magma in the narrower regions loses heat quickly and freezes. In the thicker regions, heat is lost less rapidly than in narrower regions, making the flow move faster and thermally erode the host rock. This dynamic may be initiated by the effect of the topographic stress gradients we identified in our study. In the eruptive fissures of Lakagígar and Eldgjá, Permissive domains would favour magma pressure build-up, providing more favourable conditions for the shallow fissure to exceed the aperture threshold required for thermal erosion and channel formation^44^. This induces a loop in which higher flow rates at these sites produce more heat, further melting the walls. This causes the initial continuous fissure eruption to rapidly localise into a few distinct point sources or plugs^8^, eventually resulting in sharp contrasts in the dyke width that cause the eruption to localise into sustained, isolated cylindrical cones. In Restrictive domains, with higher CSG, the higher confining stress may keep the initial fissure aperture narrow, which accelerates heat loss and favours the shallow magma to solidify, preventing voluminous cones from developing.

The process of flow focusing and thermal erosion, which explains the transition from an initial extensive fissure to localised vents, was described at several recent basaltic eruptions. During the 2014–2015 Holuhraun eruption in Iceland, active lava fountains decreased from 57 to only 10 within 5 days as dedicated magma channels were established^45^. Similarly, during the 2018 lower East Rift Zone eruption of Kilauea, initial fissures spanning hundreds of metres remained active only briefly before eruptive activity became focused at a single location within just 12 h of its reactivation^46^. During the 2021 Fagradalsfjall eruption, the initial 180-m-long fissure quickly concentrated into two neighbouring vents^6^.

Surface processes further amplify these mechanically controlled patterns. During the early phases of both eruptions, magma-water interactions played a fundamental role and produced tuff and scoria cones^30^. The final volume of these cones depends strongly on fragmentation efficiency and eruptive style: in low-elevation areas, typically corresponding to Permissive domains, water availability enhances explosive magma fragmentation. The resulting pyroclasts accumulate efficiently around the vent, building broad and voluminous tuff and scoria cones^30^.

In contrast, cones located in higher, drier sectors—typically corresponding to Restrictive domains—tend to erupt through purely magmatic fire-fountaining. Spatter clasts produced during these events fall close to the vent and often feed clastogenic lava flows rather than building large cones^30^. Steeper slopes in these elevated regions further limit pyroclast accumulation by promoting downslope transport of eruptive material. Consequently, in dry steep environments, a larger fraction of the erupted mass is transferred away from the vent as lava rather than contributing to cone growth.

Beyond hydrologic and topographic controls, the dynamics of the lava fountain and stability of the different materials that are emplaced shape the cone’s morphology. Previous work shows that the inclination of the fountain, in combination with the wind direction, frequently defines cone asymmetry, as coarse hot pyroclasts may coalesce into rootless flows near the vent and smaller cooler cinders are deposited downwind^47^. Also, some cones are shaped by early structural failures, including crater collapses and small landslides from the crater rim during its growth, triggered by, for instance, changes in magma levels or fissure widening^48^. After cone emplacement, the shape and volume of cones continue to be affected by post-eruptive instabilities. As magma drains back into the dyke or conduit during the waning stages of the eruption, cone retraction and collapse processes can be triggered^30^. Such drainage processes typically manifest as arcuate cracks, subsidence and fault-bounded notches along the rim of the crater^48^. Following this initial stabilisation, cones undergo continued geomorphological degradation. Exterior slopes tend to degrade through wind dispersal and gravitational sliding of unconsolidated scoria, whereas vent interiors are primarily affected by discrete rockfalls from oversteepened walls^49^. Finally, the overall edifice volume is modified by bulk subsidence driven by sedimentary compaction and thermal cooling contraction^49^. Such structural controls and erosion mechanisms can substantially modify the preserved cone volume and contribute to the observed variability in edifice morphology.

Taken together, these observations indicate that the relationship between topography and cone volume cannot be attributed to stress alone. Rather, the observed clustering of cone sizes along both fissures emerged from a synergy of deep structural controls (CSG-driven conduit longevity), shallow hydrologic and atmospheric conditions, lava fountain dynamics, and a combination of syn-eruptive instability and post-emplacement degradation.

Comparable dynamics occur at several other basaltic volcanic systems worldwide. At Piton de la Fournaise (La Réunion), fissures traverse a pronounced topographic gradient between the steep inner caldera and the lower outer rift zones, resulting in cone volumes that span several orders of magnitude^9^. Similar to the Restrictive and Permissive domains we describe in this study, cones formed within the steep caldera are relatively small, with median volumes of 7200 m^3^, whereas those forming on the flatter plains of the outer rift zones can exceed median volumes of over 2 million m^3^^9^. Similarly, at Mount Etna, an inverse relationship exists between vent elevation and lava flow length, reflecting the influence of topography on eruption dynamics^50^. In contrast, the East Rift Zone of Kilauea (Hawaii) provides a useful counterexample. Although this rift hosts long, segmented fissures, it develops across relatively uniform topography. While these eruptive fissures are segmented, cone volumes vary much less along individual fissures, and the spatial variability in fountaining intensity is largely attributed to internal magmatic processes such as pressure waves propagating through the dyke^24^, rather than topographic loading. The strong topographic signal observed at Lakagígar, Eldgjá and Piton de la Fournaise therefore highlights how surface relief can fundamentally reorganise the distribution of eruptive vents and the morphology of fissure eruptions.

Beyond cone volume, topography-induced stresses are also responsible for fissure segmentation. Although regional tectonic stress is recognised as the primary control on the orientation and architecture of fissure eruptions^51,52^, topographic loading and unloading can perturb the local stress field by rotating the principal stress axes^53,54^. Surface loads tend to rotate the maximum compressive stress toward the load, potentially attracting dyke propagation paths while simultaneously increasing compressive stresses that may inhibit upward magma ascent^55,56^.

These mechanisms operate at a wide range of volcanic systems. At massive shield volcanoes such as Mauna Loa, the gravitational loading of the edifice controls the development of flank rift zones^57^. Similarly, magma migration away from the central load has been invoked to explain the distribution of activity at stratovolcanoes such as Nyiragongo^14^. Conversely, large-scale surface mass redistribution from flank collapses also alters crustal stresses^12^. Analogue and numerical models, supported by field data from systems like Mount Etna^58^ and Stromboli^59^, show that collapse depressions and scarps deflect dykes and often relocate the volcanic activity^12^.

Within this framework, topographic stress gradients dictate fissure segmentation across scales. As a laterally propagating dyke encounters abrupt stress transitions—typically generated by steep mountain slopes—the resulting increase in resistance may arrest the dyke at shallow depth, producing a segmented pattern of eruptive vents at the surface. While sharp peaks in horizontal stress gradients cause regional-scale segmentation at Eldgjá, the mechanism also manifests locally at Lakagígar. Where the fissure crosses Mount Laki^33^, vents disappear along the ascending slope, reappear within local depressions, and then resume on the opposite flank (Fig. S2 in the Supplementary information). The apparent contrast between the pronounced segmentation and gaps at Eldgjá and the seemingly continuous fissure at Lakagígar may therefore partly reflect differences in observation scale rather than fundamentally different processes. In both systems, local topographic gradients seem to act as barriers to dyke propagation, controlling the fissure architecture at spatial scales proportional to the surrounding topography.

Ultimately, the identification of the topographic stress gradients as a proxy for shaping fissure eruptions has important implications for volcanic hazard assessment. Probabilistic hazard models typically rely on historical vent distributions and lineaments to map the susceptibility of new vents opening^60,61^. Our results demonstrated that stress-derived parameters can improve these forecasts. The analytical approach that we propose is, however, retrospective, as the CSG thresholds that define the Permissive and Restrictive domains were obtained with the actual observations to maximise the fit. Prior to an eruption, such tuning is not possible. However, scenario-based forecasting can be achieved by testing possible values derived from historical analogues. For instance, applying the 0.23 MPa/km threshold from Lakagígar could serve to generate probabilistic hazard maps for future dykes fed by the Grímsvötn system. Alternatively, applying the 0.23–0.46 MPa/km threshold range observed across both eruptions could define Permissive and Restrictive envelopes for the broader EVZ. These ideas of approaching the hazard assessment are, however, speculative and require validation from other cases worldwide.

Preliminary observations at recent events worldwide suggest these dynamics are directly transferable to other basaltic systems. For instance, during the 2021 Tajogaite eruption on La Palma (Canary Islands), the eruptive fissure opened on the western flank of the Cumbre Vieja ridge, aligning obliquely to the slope, producing a “chimney effect" at the vents: the highest vents acted as explosive degassing pathways, while the lower ones mainly fed lava flows^62^. Similarly, recent eruptions in the Fagradalsfjall volcanic system in Iceland demonstrated clear topographic effects. The 2022 eruptive fissure opened within the Meradalir valley and terminated abruptly against its steep valley walls^63^.

The recent eruptive crises on the Reykjanes Peninsula, including Fagradalsfjall and Sundhnúkur, have demonstrated the importance of rapid hazard assessment and early warning systems to protect local communities and critical infrastructure^64,65^. While current monitoring techniques now provide unprecedented early warnings regarding the timing of dyke intrusions, anticipating the spatial location of new fissure openings at the surface remains a major challenge^64,65^. Integrating topographic stress models into this well-established monitoring framework could improve the forecast by assessing not only the possible spatial propagation and segmentation of eruptive fissures but also the morphological variation and eruptive styles of associated vents. This work aims to inspire future research to validate our predictive stress framework in diverse tectonic regions, further refining the model for forecasting fissure propagation and vent evolution.

## Methods

### Elevation data

Regional-scale mapping of the eruptive cones and extraction of along-fissure elevation profiles were based on the 2 m × 2 m ÍslandsDEM v.1.0, accessed via the National Land Survey of Iceland (LMI). ÍslandsDEM is a spatially adjusted and robustly mosaicked derivative of the ArcticDEM dataset. The mosaic was developed through collaboration between the Icelandic Institute of Nature Research, the Icelandic Meteorological Office, and the Polar Geospatial Center (PGC). Each pixel in the ÍslandsDEM mosaic corresponds to a median elevation value derived from the multitemporal ArcticDEM data. The original ArcticDEM models were generated from sub-metre commercial stereo imagery (e.g., WorldView 1–3 and GeoEye-1) processed using SETSM photogrammetric software^66^. The satellite imagery used to generate these digital elevation models is multitemporal, typically acquired from 2012 to the present, with the oldest data dating back to 2008.

The DEMs were used to map eruptive cones along the full length of both eruptive fissures, quantify cone volumes, and analyse their distributions relative to topography. To resolve very small-scale eruption cones, a higher resolution (sub-metre) DEM was required for selected regions that were central to testing our concept and displayed a high level of complexity. To this aim, we acquired high-resolution aerial optical imagery using an uncrewed aerial vehicle (UAV) over targeted sections of the Lakagígar fissure. The drone surveys covered a total area of 6.6 km^2^, extending continuously between 64.05°N, 18.28°W and 64.09°N, 18.20°W along the fissure (see Fig. S2A in the Supplementary information for spatial coverage), which corresponds to the section between 10.8 and 16.1 km distance along the fissure in Fig. 2B, which covers the extension of Mount Laki. Data were collected using a WingtraOne Gen II fixed-wing Vertical Take-Off and Landing (VTOL) drone, equipped with a Sony Alpha 7R IV full-frame camera (61 MP sensor, 24 mm fixed focal length lens). The drone followed the terrain at 120 m above ground level, acquiring images with high overlap (70% lateral, 80% frontal). High-precision georeferencing was achieved using a dual-frequency GNSS base station and post-processing kinematics (PPK) processing, ensuring centimetre-level accuracy^67^. The PPK geotagging of the dataset was done using WingtraHub v2.19.0 and achieved a mean absolute accuracy of 2 cm horizontally and 3 cm vertically. The images were processed in Agisoft Metashape (v. 2.2) using Structure-from-Motion (SfM), producing a DEM and orthomosaic with a spatial resolution of ~20 cm/px. This resolution made it possible to map cones down to ~2 m diameter, whereas the regional 2 m resolution ÍslandsDEM constrained our detection limit to structures with diameters larger than ~10 m. This increase in resolution relative to the ÍslandsDEM was critical for identifying and mapping small-scale eruptive cones.

### Geoinformatics

We performed a geospatial analysis to quantify the relationship between the surface cones and the underlying topography using QGIS (v. 3.40.15), a freely available Geographic Information System (GIS). The GIS workflow consisted of mapping eruptive cones, calculating the cone volumes, and reconstructing the pre-eruptive topography.

#### Mapping and morphometry

To delineate the basal surfaces of the eruptive cones, we initially used an automated mapping approach, the NETVOLC algorithm^35^, on a representative subset of 40 cones along the Lakagígar fissure (Fig. S1 in the Supplementary information). The automated detection accurately delimited three isolated, highly symmetrical eruptive cones (Fig. S1A in the Supplementary information), while five were mapped with moderate precision (Fig. S1B in the Supplementary information). The remaining 32 cones (80%) were poorly defined by the algorithm due to coalescence, irregular topography, and sensitivity to input parameters like window size (Fig. S1C in the Supplementary information).

Consequently, to avoid biases and ensure consistency, we mapped the cones manually. For this, we used a combination of DEM derivatives, combining the 2-m resolution ÍslandsDEM and the 20-cm resolution drone DEM, along with optical imagery. The boundary for each eruptive cone was defined by tracing a sharp break in slope marking the transition from the steep cone flanks to the flatter surrounding terrain. We used hillshade models and slope maps with the drone orthomosaics, supplemented by high-resolution aerial imagery accessed via the Icelandic mapping portal^68^. Despite the high resolution of these datasets, manual mapping required specific criteria, introducing inherent uncertainties. First was cone coalescence, which required subjective interpolation to define individual basal boundaries. For highly elongated, coalesced structures, we used curvature changes along the crater to delineate the approximate dimensions of individual cones. Also, in cases where smaller secondary vents were superimposed on larger edifices, we chose to map the primary, larger cone. Furthermore, lava flows frequently cover parts of the cones, particularly at the lower flanks, complicating the cone base definition. However, since our statistical analysis considers order-of-magnitude variations in cone volume, these mapping uncertainties represent only minor variability and do not fundamentally alter the observed eruptive cone size distribution along the fissures.

For the cone volume calculation, we used the Volume Calculation Tool plugin^69^ in QGIS. The reference base height was determined by the average elevation of the polygon vertices, and the volume was calculated as the space between this approximated base and the digital elevation model.

#### Pre-eruptive topography reconstruction

The reconstruction of pre-eruptive topography is fundamental in volcano morphometry studies, serving multiple scientific purposes. Isolating the pre-existing surface from a digital elevation model is widely used, for instance, for calculating erupted volumes and magma flux estimations^70,71^, as well as in lava flow emplacement simulations^72^. Such reconstructions either use historical pre-eruption maps^71^, or apply interpolations using the unburied adjacent terrain and other field and stratigraphic data^70,72^. Here, we approximated the pre-eruptive topography to test topographic effects on eruptive cone volume by isolating the pre-existing basement from the mapped eruptive cones. We extracted elevation profiles along the fissures using a 1200-m-wide buffer sampled at 50 m intervals from the ÍslandsDEM (Fig. 3). To approximate the pre-eruptive topography, we processed these profiles in Python using the *SciPy* library^73^ to smooth the topographic data and apply a spatial filtering (see the section “Code availability”). Specifically, we applied the following filtering approach: (1) a sliding-window minimum filter (1 km width) was applied to remove positive anomalies corresponding to new cones; (2) pre-existing mountains (e.g., Mount Laki) were masked out to preserve their original elevation; and (3) the resulting basement profile was smoothed using a Gaussian filter (*σ* = 3 pixels, 150 m) to ensure smooth transitions. While this approach extracts localised positive anomalies (i.e., eruptive cones), it does not fully remove the lava flows cover. The average thickness of lava fields at both Laki and Eldgjá is typically <25 m^30,39^. The main topographic features here have elevation contrasts of hundreds of metres, representing an order of magnitude difference, which implies that the models would not vary dramatically. Furthermore, because lava flows accumulated preferentially in depressions, an approach that removes such lava thickness from our pre-eruptive models would result in deeper valleys, steeper terrain, and more accentuated gradients in topography, which would further amplify the topographic stress gradients and further support our observations. This means that our topographic stress models are conservative estimations, and thus, the mechanical controls identified are robust against uncertainties from lava infill.

### Topographic stress models

We used analytical solutions for uniform vertical strip loads applied on an elastic half-space^13,74^ to model the crustal stress induced by topography. The 2D pre-eruptive elevation profiles (see the section “Pre-eruptive topography reconstruction”) were discretised into rectangular load elements with a defined width of 50 m and a height equal to the local elevation relative to the regional base level (lowest point along each profile). We assumed a constant density of 2700 kg/m^3^^75^ and a Poisson’s ratio (*ν*) of 0.25, consistent with crustal models of the region^3^.

Stress components were computed on a regular grid (50 m spacing) extending to 3 km depth by summing the contributions of all topographic load elements at each node. While the full stress tensor was calculated, our focus was on the out-of-plane stress component (*S*_*yy*_), which acts perpendicular to the dyke plane (striking ~ NE–SW and dipping vertically). Assuming plane strain conditions, this component is defined as *S*_*yy*_ = *ν*(*S*_*xx*_ + *S*_*zz*_), where the (*x*, *z*) plane coincides with the dyke plane (*x* being horizontal, positive NE-wards, *z* being vertical, positive downwards). We evaluated the magnitude and direction of the maximum *S*_*yy*_ gradient, considering that magma flow is inhibited in the direction of increasing confining stress. The entire stress field and spatial gradients were computed through array operations using the *NumPy* library^76^ in Python (see the section “Code availability”).

### Statistical analysis

We implemented quantitative statistical workflows on our data using Python (see the section “Code availability”). We used *Pandas* to manipulate the dataset^77^, *Scikit-learn* for machine learning workflows^78^, including the *K*-means clustering and the receiver operating characteristic (ROC) analyses, *SciPy* for the peak-detection algorithms^73^, and *Matplotlib*^79^ and *Seaborn*^80^ for data visualisation. The aim was to determine if stress gradients can be used to distinguish between sectors hosting large-volume cones, small-volume cones, or non-eruptive gaps in the fissures. Our approach involved an initial exploratory analysis of the correlations between stress parameters and fissure morphology and architecture, followed by the validation of predictive models at each fissure (Lakagígar and Eldgjá) based on actual observations. We applied a consistent modelling framework to both fissures, adapting it to their specific geomorphological characteristics: while the analysis of Lakagígar focused on cone volume distribution, the analysis of Eldgjá also addressed the prediction of fissure segmentation (gaps) alongside cone volumes. Our test hypothesis was that both (cone volumes and fissure segmentation) reflect a common mechanism: the control on magma transport exerted by crustal stress gradients.

#### Exploratory phase and metric selection

We performed an exploratory analysis to identify the stress parameters associated with eruptive metrics (cone volume and segmentation). We focused on testing three potential metrics: the horizontal stress gradient (∣d*S*_*yy*_/d*x*∣), the absolute vertical stress gradient (∣d*S*_*yy*_/d*z*∣), and the combined stress gradient (CSG). The CSG is defined as the sum of both orthogonal stress gradients (CSG = ∣d*S*_*yy*_/d*x*∣ + d*S*_*yy*_/d*z*), which considers the absolute value for the horizontal gradient, as upward magma propagation will be laterally deflected by high horizontal stress gradients regardless of polarity. In contrast, the sign of the vertical component is relevant as it allows us to distinguish between vertical gradients that oppose buoyancy (with a positive sign, increasing compression upwards) and those favouring it (with a negative sign, decreasing compression upwards).

Comparative analysis (kernel density estimates, ROC curves; Supplementary Figs. S3–S6) revealed that the CSG generally provided the strongest discrimination between large and small cones, particularly for the Lakagígar dataset (Supplementary Figs. S3, S5). In Eldgjá, a similar trend was observed (Supplementary Figs. S4, S6), even though the statistical analyses did not acknowledge the actual match between restrictive regions and fissure gaps that our CSG model generally provides (Fig. 5F).

#### Cone volume prediction

We developed a binary classification model to predict the location of large-volume eruptive cones. The workflow consisted of three steps, applied to both systems separately: (1) Defining large vs. small cones: we began by applying a *K*-means algorithm (*k* = 2) to the log-cone volumes. This approach defined a cutoff that separated the population into small and large cone volume clusters, which corresponded to 10^3.8^ m^3^ for the Lakagígar system and 10^5.5^ m^3^ for Eldgjá. (2) We calculated ROC curves to evaluate the predictive capacity of the chosen stress metric, CSG, to discriminate between the two clusters, which is represented by the area under the curve (AUC). We used Youden’s *J* index (*J* = Sensitivity + Specificity−1)^81^ to identify the optimal CSG threshold (that maximises this index). This threshold divides the fissure into two stress domains: a Permissive domain favouring volume growth (below the CSG threshold), and a Restrictive domain inhibiting volume growth (above the CSG threshold). (3) Projection of the Permissive and Restrictive domains was calculated for each system by applying the threshold along the fissures CSG. We validated the model by quantifying the percentage of large cones successfully captured within Permissive domains (Sensitivity) and the percentage of cones in Restrictive regions that were small.

#### Fissure segmentation prediction

Eruption gaps and fissure segmentation are major structural features in the architecture of fissure eruptions observed globally^3,4,51^. We additionally focused on finding the best stress metric for predicting fissure gap occurrence. In fact, the previous workflow, based on the correlation between cone volumes and CSG values, lacked statistical constraints where cones were absent (i.e., fully inhibited). As fissure segmentation at Eldgjá is spatially associated with the sharpest lateral changes in the confining pressure, we developed a peak-detection algorithm based on the signed horizontal stress gradient d*S*_*yy*_/d*x*. We extracted the along-strike stress gradient profile by averaging the top 0.5 km of the topographic stress model. The topographic barriers that induce segmentation (i.e., E1–E4 mountains crossing the fissure) are bounded by opposite slopes (positive to end a fissure segment, negative to start one). Such sharp slopes induce a characteristic signal of alternating large positive and negative peaks in the horizontal stress gradient, corresponding to the increasing and decreasing confining stress variations along the horizontal direction. To identify them, we calculated the 90th percentile threshold of the gradient magnitude and applied a peak finder algorithm (SciPy) to detect successive opposite peak values. Eruptive gaps were predicted as the intervals between pairs of alternating peaks. By comparing the predicted gaps against the real segmentation, we assessed the model’s accuracy.

## Supplementary information

## Data Availability

The drone data and shapefiles generated and analysed during the current study are available in the GFZ Data Services repository^82^.

## Code availability

The Python code used for the pre-eruptive topography reconstruction, topographic stress modelling and statistical analyses is openly available. The code, together with corresponding input data files for the Lakagígar case study, is provided as a Compute Capsule through Code Ocean^83^.
