# Supplementary material for: Topographic stress controls fissure segmentation and eruptive cone formation during the Laki and Eldgjá eruptions
Source: Commun Earth Environ. 2026 Sep 2;7(1):719. doi: 10.1038/s43247-026-03980-4 (PMC13538144; doi:10.1038/s43247-026-03980-4)
Supplement: Supplementary file 1 [file 43247_2026_3980_MOESM1_ESM.pdf]

nature portfolio

Peer Review File

Topographic stress controls fissure segmentation and eruptive cone formation during the Laki and Eldgjá eruptions

Corresponding Author: Ms Maria Hurley

This file contains all reviewer reports in order by version, followed by all author rebuttals in order by version.

Version 0:

Reviewer comments:

Reviewer #1

(Remarks to the Author)
Review of “Topographic stress controls fissure segmentation and eruptive cone formation during the Laki and Eldgjá eruptions” by Hurley et al.

This paper describes how topographic stresses can inhibit cone formation, using examples in Iceland as demonstrative case studies. This work builds on existing work and is clearly novel with broad implications for fissures globally. This suits the scope and breadth of Communications Earth & Environment and would be an excellent addition to the journal.
I found the paper to be very well written and clear. This is an excellent manuscript and was a joy to read. There are certainly points of clarification that would benefit from additional explanation, but these are minor. As such, I have officially recommended minor revisions. I hope the authors find my comments constructive.
Minor suggestions, noted by the corresponding line number:
63-66: You have mentioned Afar and other Iceland examples. It would be useful to add at least two more where the tectonic setting may be quite different so that your analysis is more globally relevant. You could refer to the Kilauea example i.e., ref 16 here. By the way, that reference needs editing.
It would be useful to refer to the ongoing Reykjanes fissures to highlight the importance of your work in both an operational forecasting sense, but also to reduce impacts if a predictive model is able to be developed.
An attribute that is not explored, but should be mentioned, is time. For example, are these cones generated sequentially, or are they more chaotic in their pattern? This is important because the timing of cone formation along the fissure may play a role in affecting the stress state, along with the topographic loading.
73: I would refer to these examples to bolster your point that I make above.
77: by largest, do you mean by eruptive volume, size of fissure? Please specify
Note: this Introduction is excellent and sets the scene very clearly.
97: insert “respectively” after tephra
98: the eruption indicates that you are discussing one of the two. Which one? Change to “After the start of each eruption, the opening feeder dykes migrated north-…”
119: cone not cones
125: southwest not Southwest
173: Looking at your CSG analysis, the correlations look strong. I am left wondering why an analysis of 0-3km wasn’t attempted. This would be useful to see where the CSG would start to fail. Without this, I cannot assess whether this 0-2km is optimal or not.
Limitations: One thing to consider is the evolution of the magma within the fissure. This is not accounted for in your analysis. You have been clear in the limitations of your model, which I completely agree with. However, magma will be potentially cooling, crystallizing, and therefore, the viscosity will be changing throughout flow. This may mean that some zones of high stress gradients may be areas for magma ascent whereas others may not. It is unclear from the data you present that this is the case in either example, but it may be worth mentioning here. This point relates to your sensitivity analysis and it could be linked to your assessment that 0-2 km provides a correlation i.e., fissure flow along a sill.
252: does not rather than do not
334-337: are you implying that the topographic effect may control the width of dykes? As written, that could be seen as a reasonable extension of the description provided.
336: heat is lost in thicker dykes, but much less rapidly. So yes, you are right that thicker dykes retain heat, but only relative to their thinner counterparts.

338: "…we identified in the our study…" - remove the
349-354: do you have other examples to describe and demonstratable cases?
357-367: does this assessment re: water availability and elevation align with the results of ref 23? If you could tie these together, that would be very helpful
387: describe rather than described – keep the tenses the same
417-433: this text block is simply a summary of already presented text. Suggest deleting.
References: Ref 12 is unfinished. In fact, many are poorly formatted and need editing

Reviewer #2

(Remarks to the Author)
The authors present an intriguing study regarding the controlling processes for volcanic cones formed during the Laki and Eldgja eruptions. The Figures are excellently made and clearly present the results of the study. The Discussion section explains the limitations of the study and contextualizes future relevance of the work. The data and methods are mostly explained in a manner that future studies could reproduce them. My feedback on the manuscript is relatively minor and consists of a few moderate comments for the authors to consider and line edits. Overall, the manuscript is well written and presented. -Ian T. W. Flynn

Moderate Comments:
• In section 3.6 the authors acknowledge that their study is retrospective and thus not applied during an active eruption. I agree with the author's discussion that this approach could be useful in some hazard assessments, but additional detail is needed. Specifically, how would the methods used here be used to support future hazard assessment? The approach presented does not appear to be feasible or relevant once an eruption has started. Therefore, would it be applied pre-eruption to assess where cones may form? Additional text is required in this section to make it clearer how the methods used in this study could be applied to future volcanic hazards.

• Within the discussion section (Section 3) there needs to be additional details acknowledging the other processes that can influence cone formation. Stability of the emplaced cone material can influence the final morphology, orientation of the fountaining can change the shape of the cone, and as observed currently at Kilauea the wind direction can influence the deposition of scoria. I provide a few example references below, but this aspect of the manuscript should be expanded.
o Calvari, S., & Pinkerton, H. (2004). Birth, growth and morphologic evolution of the 'Laghetto'cinder cone during the 2001 Etna eruption. Journal of Volcanology and Geothermal Research, 132(2-3), 225-239.
o Head III, J. W., & Wilson, L. (1989). Basaltic pyroclastic eruptions: influence of gas-release patterns and volume fluxes on fountain structure, and the formation of cinder cones, spatter cones, rootless flows, lava ponds and lava flows. Journal of volcanology and geothermal research, 37(3-4), 261-271.

• In multiple locations within Section 4 the authors refer to the use of python libraries. This is useful but requires additional information to be relevant. Specifically, please state the analysis that these libraries were used to complete. Each library contains numerous processes and options, and simply stating the library used without context is unhelpful.

Line Edits:
Line 63-66: Scoria/cinder and ash cones are the same features. Spatter cones form through the agglutination of fragmented lava. This sentence is a bit of an oversimplification as not all these cones are symmetrical, and their morphology does differ between the subsets (i.e., scoria vs spatter). Two recommendations for this sentence: (1) remove ash cone from the sentence; (2) add supporting references to the sentence.
Line 116-121: Is there a Figure or table that supports these results?
Line 159-160: Can you add a range of slope values with "gentler slopes"?
Line 213: Can you please say what exactly is the percent? Saying "almost 100%" could be a lot of values and may be misinterpreted by the reader.
Line 220: Can you please provide the exact count of cones in the catalog?
Line 224-239: I appreciate the authors acknowledging the potential observation bias of the study. However, cones smaller than what is identifiable in a 2 m/pixel should be minimal. Acknowledging the limitations of the study is important, but the authors could probably soften the language in these sentences to recognize that smaller cones are unlikely to be present.
Line 375-377: Should there also be a few sentences about post emplacement erosion? There has been a lot of work on how the shape of cones/vents change after emplacement, see example references below.
• Sutton, S. S., Richardson, J. A., Whelley, P. L., Scheidt, S. P., & Hamilton, C. W. (2024). Degradation of the 2014–2015 Holuhraun vent-proximal edifice in Iceland. Bulletin of Volcanology, 86(4), 37.
• van Wees, R. M., O'Hara, D., Kereszturi, G., Paguican, E., Grosse, P., Lahitte, P., & Kervyn, M. (2026). Long-term erosion dynamics and morphological evolution of composite volcanoes: Insights from analogue modelling. Journal of Volcanology and Geothermal Research, 108654.
Line 444: Remove significantly from this sentence. Your results demonstrate potential to improve hazard modeling but if that improvement is significant is not supported.
Line 467: Please specify what kind of regional analysis.
Line 473: Extra space at the end of this sentence.
Line 473-475: Somewhere in this first paragraph could you add a sentence for when this dataset was collected. I realize that your study area is well before any of these DEMs were generated but this may be relevant to future researchers trying to

reproduce your study or build on it.
Line 482-485: On one of the figures or in a new supplemental one, could you please display the area that the drone data covers? This will help readers clearly understand the spatial coverage of this dataset.
Line 493: Please report the vertical and horizontal accuracy of this dataset.
Line 580-581: What specific statistical analysis did you perform? Stating the python libraries, you used is insufficient. See major comment.

Open Access This Peer Review File is licensed under a Creative Commons Attribution 4.0 International License, which permits use, sharing, adaptation, distribution and reproduction in any medium or format, as long as you give appropriate credit to the original author(s) and the source, provide a link to the Creative Commons license, and indicate if changes were made.
In cases where reviewers are anonymous, credit should be given to 'Anonymous Referee' and the source.
The images or other third party material in this Peer Review File are included in the article's Creative Commons license, unless indicated otherwise in a credit line to the material. If material is not included in the article's Creative Commons license and your intended use is not permitted by statutory regulation or exceeds the permitted use, you will need to obtain permission directly from the copyright holder.
To view a copy of this license, visit https://creativecommons.org/licenses/by/4.0/

Line numbers indicated in the responses always refer to the marked up document "Main_Revised_MarkedUp.pdf"

Reviewer 1

Comment 1.1:

You have mentioned Afar and other Iceland examples. It would be useful to add at least two more where the tectonic setting may be quite different so that your analysis is more globally relevant. You could refer to the Kilauea example i.e., ref 16 here. By the way, that reference needs editing. See line 73, those examples can be used.

Response:

We thank the reviewer for this suggestion. We agree that introducing examples from diverse tectonic settings early in the manuscript highlights the global relevance of our findings. We have modified the Introduction (**Lines 45-47**) to include the Kilauea (Hawaii) and Piton de la Fournaise (La Réunion) examples, moving these references up from the previous line 73, as suggested.

Additionally, we have corrected the formatting of Reference 16 in the bibliography and also others that needed editing.

Comment 1.2:

It would be useful to refer to the ongoing Reykjanes fissures to highlight the importance of your work in both an operational forecasting sense, but also to reduce impacts if a predictive model is able to be developed.

Response:

We agree with the reviewer, the ongoing eruptive events on the Reykjanes Peninsula are excellent examples of the need for spatial forecasting to mitigate infrastructural and societal impacts. Following this suggestion, we have now included the recent 2021–2026 Reykjanes eruptions as a key example in the Introduction (**Lines 44-45**).

The clean text, considering the changes regarding Comment 1.1 and 1.2, now reads: **(Lines 39-47)**
"Similar patterns of localized vent formation along otherwise continuous fissure systems are observed in Icelandic rift zones, such as the 1975–1984 Krafla [5] and 2014–2015 Bárðarbunga–Holuhraun [3] events, and more recently in the 2021–2026 eruptions on the Reykjanes Peninsula [6, 7]. Similar segmentation occurs in other tectonic settings, including the oceanic shield volcanoes of Kilauea in Hawaii [8], and Piton de la Fournaise in La Réunion [9]."

Furthermore, to address the forecasting aspect, we have updated the Discussion on the last part where we discuss the **implications for hazard assessment**, Lines 597-606 to explicitly discuss how integrating our topographic stress models complements existing operational frameworks. The clean text now reads (**Lines 597-606**):

"The recent eruptive crises on the Reykjanes Peninsula, including Fagradalsfjall and Sundhnúkur, have demonstrated the importance of rapid hazard assessment and early warning systems to protect local communities and critical infrastructure [64, 65]. While

current monitoring techniques now provide unprecedented early warnings regarding the timing of dike intrusions, anticipating the spatial location of new fissure openings at the surface remains a major challenge [64, 65]. Integrating topographic stress models into these well-established monitoring framework could improve the forecast by assessing not only the possible spatial propagation and segmentation of eruptive fissures but also the morphological variation and eruptive styles of associated vents."

Comment 1.3:

An attribute that is not explored, but should be mentioned, is time. For example, are these cones generated sequentially, or are they more chaotic in their pattern? This is important because the timing of cone formation along the fissure may play a role in affecting the stress state, along with the topographic loading.

Response:

We agree that time is a crucial factor. Based on historical accounts and tephra stratigraphy, cone formation at both Lakagígar and Eldgjá was sequential, generally propagating from southwest to northeast. We have addressed this in the Discussions, together with other limitations of the models, by acknowledging that our static models do not capture this temporal evolution, and discussing how progressive cone growth dynamically alters the topographic stress field over time. The text now reads **(Lines 369-377):**

"Finally, our models are static and do not account for the temporal evolution of the eruptions. At both Lakagígar and Eldgjá, cone formation was sequential following discrete eruptive episodes generally propagating from southwest to northeast [30, 39]. This implies that the stress field varies with time as dykes are emplaced and cones grow, dynamically affecting subsequent magma focusing and segmentation. Furthermore, as magma propagates laterally away from the source, processes like crystallization and heat loss alter its rheology (e.g., increasing its viscosity). This means that applying a uniform stress threshold for defining Permissive and Restrictive domains along the entire fissure is a limitation."

Comment 2:

This study investigates magma-topography interactions by analyzing two of the Earth's largest basaltic events:" by largest, do you mean by eruptive volume, size of fissure? Please specify

Response:

We agree that the term "largest" is ambiguous. In order to clarify that we are referring to the total magma output, we have rephrased this to specify that these are the most "volumetrically productive" historical basaltic events.

The revised sentence (Lines 89–92) now reads:

Line numbers indicated in the responses always refer to the marked up document “Main_Revised_MarkedUp.pdf”

"This study investigates magma-topography interactions by analyzing two of the Earth’s most volumetrically productive historical basaltic events: the Eldgjá (~939–940) and Laki (1783–1784) eruptions.”

Comment 3 (various):

L97: insert “respectively” after tephra

L98: the eruption indicates that you are discussing one of the two. Which one? Change to “After the start of each eruption, the opening feeder dykes migrated north-…”

L119: cone not cones

L125: southwest not Southwest

Response:

Accepted and changes applied.

Comment 4:

One thing to consider is the evolution of the magma within the fissure. This is not accounted for in your analysis. You have been clear in the limitations of your model, which I completely agree with. However, magma will be potentially cooling, crystallizing, and therefore, the viscosity will be changing throughout flow. This may mean that some zones of high stress gradients may be areas for magma ascent whereas others may not. It is unclear from the data you present that this is the case in either example, but it may be worth mentioning here. This point relates to your sensitivity analysis and it could be linked to your assessment that 0-2 km provides a correlation i.e., fissure flow along a sill.

Response:

We agree that as magma propagates laterally, cooling and crystallization change its rheology (e.g., increasing viscosity) which implies that using a single uniform threshold is indeed a simplification and a limitation. Following the comment, we have improved section 3.2. and added the following sentences in **Lines 374-377**

“Furthermore, as magma propagates laterally away from the source, processes like crystallization and heat loss alter its rheology (e.g., increasing its viscosity). This means that applying a uniform stress threshold for defining Permissive and Restrictive domains along the entire fissure is a limitation.”

Comment 5:

Looking at your CSG analysis, the correlations look strong. I am left wondering why an analysis of 0-3km wasn't attempted. This would be useful to see where the CSG would start to fail. Without this, I cannot assess whether this 0-2km is optimal or not.

Response:

We followed the suggestion and extended our sensitivity analysis up to 0-3 km and hence tested the whole extension of our stress models. As shown in the updated Supplementary Figures (Figs. S3 to S6), the results are still the same also with this target depth, which further demonstrates that using a depth-averaged metric from the surface to the target depth, like the CSG, preserves the shallow topographic signal regardless of the target depth chosen. We have added to the manuscript the following sentence (**Lines 226-228**)

*"To capture the influence of shallow crustal stress on magma transport, we calculated the CSG parameter by averaging the stress values within the uppermost 0.5 km. Our sensitivity analysis confirmed that results remain stable considering depth ranges between 0-0.2 km to **0-3.0** km (Figs. S3, S4). **This demonstrates that using a depth-averaged metric from the surface to the target depth, like the CSG, preserves the shallow topographic signal regardless of the target depth chosen."***

Comment 6:

"Small variations in the initial thickness of the fissure amplify over time because magma in the narrower regions loses heat quickly and freezes. In the thicker regions, heat is retained, making the flow move faster and can thermally erode the host rock." Are you implying that the topographic effect may control the width of dykes? As written, that could be seen as a reasonable extension of the description provided.

also

Heat is lost in thicker dykes, but much less rapidly. So yes, you are right that thicker dykes retain heat, but only relative to their thinner counterparts.

Response:

We thank the reviewer for pointing this out. Regarding the topographic effect: we do not mean to suggest that the dyke thickness is controlled by topography. Rather, topography may modulate the near surface aperture of the fissure (and therefore we changed the word *thickness* for *aperture*). Where the topographic stresses are lower, the magma overpressure may locally allow the fissure to open more, which in turn promotes thermal erosión and vent localization.

Second, regarding the heat loss, we agree with the reviewer's recommendation and have refined the text accordingly, specifically adjusting the terminology by avoiding the wording "dyke thickness" and rather mentioning fissure aperture and shallow magma.

The updated text now reads as follows (**Lines 433-449**):

Line numbers indicated in the responses always refer to the marked up document "Main_Revised_MarkedUp.pdf"

*"Small variations in the initial **aperture** of the fissure amplify over time because magma in the narrower regions loses heat quickly and freezes. In the thicker regions, **heat is lost less rapidly than in narrower regions**, making the flow move faster and thermally erode the host rock. This dynamic may be initiated by the effect of the topographic stress gradients we identified in our study.*

*In the eruptive fissures of Lakagígar and Eldgjá, Permissive Domains would favor magma pressure build-up providing more favorable conditions for the **shallow fissure to exceed the aperture threshold** required for thermal erosion and channel formation [43]. This induces a loop in which higher flow rates at these sites produce more heat, further melting the walls. This causes the initial continuous fissure eruption to rapidly localize into a few distinct point sources or plugs [8], eventually resulting in sharp contrasts in the dyke width that cause the eruption to localize into sustained, isolated cylindrical cones. In Restrictive Domains, with higher CSG, the higher confining stress may keep the **initial fissure aperture** narrow, which **accelerates** heat loss and favors the **shallow magma** to solidify, preventing voluminous cones from developing. "*

Comment 7*:*

"The process of flow focusing and thermal erosion, explaining the transition from an initial "curtain of fire" into localized vents [40], has been observed at recent fissure eruptions. During the 2014–2015 Holuhraun fissure eruption in Iceland, [41] described (...)"

do you have other examples to describe and demonstratable cases?

Response:

We thank the reviewer for the suggestion of broadening the cases. We have updated the manuscript to include two more examples, from the following works:

-Pedersen, G. B., Belart, J. M., Óskarsson, B. V., Gudmundsson, M. T., Gies, N., Högnadóttir, T., ... & Oddsson, B. (2022). Volume, effusion rate, and lava transport during the 2021 Fagradalsfjall eruption: Results from near real-time photogrammetric monitoring. *Geophysical Research Letters*, *49*(13), e2021GL097125.

-Neal, C. A., Brantley, S. R., Antolik, L., Babb, J. L., Burgess, M., Calles, K., ... & Damby, D. (2019). The 2018 rift eruption and summit collapse of Kīlauea Volcano. *Science*, *363*(6425), 367-374.

The text now reads as follows: (**Lines 450-461**):

"The process of flow focusing and thermal erosion, which explains the transition from an initial extensive fissure to localized vents, was described at several recent basaltic eruptions. During the 2014-2015 Holuhraun eruption in Iceland, active lava fountains decreased from 57 to only 10 within five days as dedicated magma channels were established [44]. Similarly, during the 2018 lower East Rift Zone eruption of Kilauea, initial fissures spanning hundreds of meters remained active only briefly before eruptive activity became focused at a single

location within just 12 hours of its reactivation [45]. During the 2021 Fagradalsfjall eruption, the initial 180-meter-long fissure quickly concentrated into two neighboring vents [6].”

Comment 7*:*

does this assessment re: water availability and elevation align with the results of ref 23 (Moreland, 2017). If you could tie these together, that would be very helpful.

Response:

We thank the reviewer for the suggestion. While Moreland (2017) details phreatomagmatism at Eldgjá, it relates to magma ice interaction rather than elevation-driven groundwater or rivers. To avoid mixing subglacial effects with topography-controlled groundwater effects, we opted not to integrate this reference in this context. We removed our previous sentence about Eldgjá’s rivers **(Lines 468-470)** and instead, we kept the citation Thordarson and Self (1993) which indeed links lower elevations and groundwater availability to phreatomagmatic cone formation at Lakagígar.

Comment 8: *417-433: this text block is simply a summary of already presented text. Suggest deleting.*

Response:

We agree that the original paragraph was repetitive. Following the reviewer’s suggestion, we have deleted the summary of our results. However, we preserved the cross-scale comparison, condensing the text to focus directly on how topographic stress gradients drive regional segmentation at Eldgjá and local segmentation at Mount Laki (supported by Supplementary Fig. S2).

The revised text (Lines 544-568) now reads as follows:

“Within this framework, topographic stress gradients dictate fissure segmentation across scales. As a laterally propagating dyke encounters abrupt stress transitions—typically generated by steep mountain slopes—the resulting increase in resistance may arrest the dyke at shallow depth, producing a segmented pattern of eruptive vents at the surface. While sharp peaks in horizontal stress gradients cause regional scale segmentation at Eldgjá, the mechanism also manifests locally at Lakagígar. Where the fissure crosses Mount Laki [32], vents disappear along the ascending slope, reappear within local depressions, and then resume on the opposite flank (Supplementary Fig. S2). The apparent contrast between the pronounced segmentation and gaps at Eldgjá and the seemingly continuous fissure at Lakagígar may therefore partly reflect differences in observation scale rather than fundamentally different processes. In both systems, local topographic gradients seem to act as barriers to dyke propagation, controlling the fissure architecture at spatial scales proportional to the surrounding topography.”

Line numbers indicated in the responses always refer to the marked up document "Main_Revised_MarkedUp.pdf"

Reviewer 2

Comment 1:

Scoria/cinder and ash cones are the same features. Spatter cones form through the agglutination of fragmented lava. This sentence is a bit of an oversimplification as not all these cones are symmetrical, and their morphology does differ between the subsets (i.e., scoria vs spatter). Two recommendations for this sentence: (1) remove ash cone from the sentence; (2) add supporting references to the sentence.

Response:

We thank the reviewer for pointing this out. Following both recommendations, we have removed "ash cones" and added appropriate references. We have also expanded the test to clearly distinguish between the formation of scoria cones and spatter cones, and corrected the assumption for symmetry, explicitly explaining the main factors driving such asymmetry.

The revised text now reads: (**Lines 67-85**)

"*As a result, the geometry and spatial arrangement of volcanic landforms serve as a permanent record of subsurface dyke dynamics. At basaltic fissure eruptions, relatively small, steep-sided volcanic landforms develop, mainly scoria cones and spatter cones [e.g., 19, 20]. Scoria cones form from the accumulation of loose, cooler pyroclasts during Strombolian activity, while spatter cones develop during Hawaiian-style fountaining through the rapid accumulation and agglutination of hot and fluid lava clots [20, 21]. These landforms are often asymmetric, driven by factors such as strong wind directions during deposition, slopes and eruption dynamics [19, 21, 22]. Furthermore, their geometry can provide a high-resolution proxy for eruptive energy and magma stalling locations [23]. For simplicity, we refer to all these monogenetic features herein as eruptive cones. This surface-subsurface link is further supported by observations of active eruptions, where surface deformation patterns from geodetic data (e.g., GNSS, InSAR) correlate directly with the volume and geometry of the feeding intrusion [4, 24].*"

Comment 2:

"In contrast, the central sector, located around Mount Laki, corresponds to the maximum pre-eruptive elevation and exhibits a marked reduction in eruptive cones volume, characterised by clusters of small cones (<10^2m3) and minor discontinuities. Finally, the northeastern extension represents an intermediate scenario, where moderate elevations host cones of intermediate to large volumes."

Is there a Figure or table that supports these results?

Response:

We thank the reviewer for pointing this out. These regional observations are directly supported by Figure 2B, which displays the elevation profile of the Lakagígar fissure overlaid with the mapped cones (scaled and color-coded by volume). Furthermore, the specific

morphological details of the central sector around Mount Laki, where the fissure exhibits a marked reduction in cone volume and minor discontinuities, are additionally supported by Supplementary Figure S2.

The revised text **(Lines 152-159**) now reads:

“The fissure segment and cone alignments located to the southwest of Mount Laki lie at the lowest elevations and host the most voluminous cones (> 10^5 m^3) (Fig. 2B). In contrast, the central sector, located around Mount Laki, corresponds to the maximum pre-eruptive elevation and exhibits a marked reduction in eruptive cone volume, characterised by clusters of small cones (< 10^2 m^3) and minor discontinuities (Fig. 2B; Supplementary Fig. S2). Finally, the northeastern extension represents an intermediate scenario, where moderate elevations host cones of intermediate to large volumes (Fig. 2B).”

Comment 3:

Can you add a range of slope values with “gentler slopes”?

Response:

We thank the reviewer for this suggestion. We extracted the slope values directly from the pre-eruptive elevation profiles used in our models. Our data shows that the "steep slopes" responsible for arresting the fissure at Eldgjá (at the exact onset of the gaps) range between **6° and 19°**. In contrast, the "gentler slopes" along Lakagígar average only **0.7°**, with an absolute maximum of **7°**. This is why at Lakagígar, topography restricts cone growth but is generally insufficient to completely arrest magma propagation and create large spatial gaps. We have updated the text and included these slope ranges.

The text now reads (**Lines 191-200**)

“The second key trend relates to the eruption gaps observed by a lack of cones at Eldgjá (Fig. 3B). Here, the steep slopes bounding topographic highs (ranging between 6° and 19° at the onset of the gaps) induced sharp horizontal changes in the stress gradient at depth, locally reaching 2 MPa/km on the SW slope of E4. These stress gradient peaks, associated with high topographic loading and high stress magnitudes, spatially coincided with the segmentation sites, i.e., locations where the eruptive fissure is interrupted by a spatial shift, sometimes accompanied by a change in orientation. In contrast, the gentler slopes at topographic highs in Lakagígar (averaging 0.7° and reaching a maximum of 7° along the profile, Fig. 3A), resulted in significantly lower gradients that remain below 1 MPa/km.”

Comment 4:

Can you please say what exactly is the percent? Saying “almost 100%” could be a lot of values and may be misinterpreted by the reader.

Response:

We have changed the text accordingly to 99% which is the real value **(see Line 282)** (only one cone centroid in a total of 105 cones analyzed falls within a predicted gap).

Comment 5:

“... allowing the construction of a database of over 300 eruptive cones…”

Can you please provide the exact count of cones in the catalog?

Response:

We have changed to 331 cones to be more precise (**Line 302**).

Comment 6:

I appreciate the authors acknowledging the potential observation bias of the study. However, cones smaller than what is identifiable in a 2 m/pixel should be minimal. Acknowledging the limitations of the study is important, but the authors could probably soften the language in these sentences to recognize that smaller cones are unlikely to be present.

Response:

We appreciate the comment from the reviewer and have accordingly softened the statement by adding the following sentence (Lines 303-312)

“While the drone-derived DEM (~20 cm/px) was useful to identify cones with volumes as small as 1 m3 in targeted areas, the regional ÍslandsDEM (2 m/px) imposes a higher minimum detection limit. ***However, such smaller structures are expected to be minimal and should not significantly change our results.”***

Comment 7:

Within the discussion section (Section 3) there needs to be additional details acknowledging the other processes that can influence cone formation. Stability of the emplaced cone material can influence the final morphology, orientation of the fountaining can change the shape of the cone, and as observed currently at Kilauea the wind direction can influence the deposition of scoria. I provide a few example references below, but this aspect of the manuscript should be expanded.

- Calvari, S., & Pinkerton, H. (2004). Birth, growth and morphologic evolution of the ‘Laghetto’cinder cone during the 2001 Etna eruption. Journal of Volcanology and Geothermal Research, 132(2-3), 225-239.

- Head III, J. W., & Wilson, L. (1989). Basaltic pyroclastic eruptions: influence of gas-release patterns and volume fluxes on fountain structure, and the formation of cinder cones, spatter

cones, rootless flows, lava ponds and lava flows. Journal of volcanology and geothermal research, 37(3-4), 261-271.

We thank the reviewer for highlighting these important factors. We completely agree that individual cone formation and initial morphology are strongly modulated by eruptive dynamics at the surface. We have expanded this part of the discussion to explicitly discuss how fountain inclination, wind-induced scoria transport, and the mechanical stability of freshly emplaced material influence cone growth and asymmetry.

The text now reads: (**Lines 478-483**)

"Beyond hydrologic and topographic controls, the dynamics of the lava fountain and stability of the different materials that are emplaced shape the cone's morphology. Previous work shows that the inclination of the fountain, in combination with the wind direction, frequently define cone asymmetry, as coarse hot pyroclasts may coalesce into rootless flows near the vent and smaller cooler cinders are deposited downwind [e.g., 46]. Also, some cones are shaped by early structural failures including crater collapses and small landslides from the crater rim during its growth triggered by, for instance, changes in magma levels or fissure widening [e.g., 47]."

Comment 9:

Should there also be a few sentences about post emplacement erosion? There has been a lot of work on how the shape of cones/vents change after emplacement, see example references below.

- Sutton, S. S., Richardson, J. A., Whelley, P. L., Scheidt, S. P., & Hamilton, C. W. (2024). Degradation of the 2014–2015 Holuhraun vent-proximal edifice in Iceland. Bulletin of Volcanology, 86(4), 37.

- van Wees, R. M., O'Hara, D., Kereszturi, G., Paguican, E., Grosse, P., Lahitte, P., & Kervyn, M. (2026). Long-term erosion dynamics and morphological evolution of composite volcanoes: Insights from analogue modelling. Journal of Volcanology and Geothermal Research, 108654.

Response:

We appreciate this valuable suggestion. Beyond emplacement dynamics, long-term morphological evolution and post-emplacement degradation play a critical role in shaping the final volumes of volcanic cones. We have added dedicated sentences in the Discussion addressing magma drainage processes, arcuate subsidence cracks, gravitational sliding of unconsolidated scoria, and bulk volume reduction driven by thermal cooling contraction and compaction.

The text now reads: (**Lines 486-500**)

"After cone emplacement, the shape and volume of cones continues to be affected by post-eruptive instabilities. As magma drains back into the dyke or conduit during the waning

stages of the eruption, cone retraction and collapse processes can be triggered [30]. Such drainage processes typically manifest as arcuate cracks, subsidence and fault-bounded notches along the rim of the crater [47]. Following this initial stabilization, cones undergo continued geomorphological degradation. Exterior slopes tend to degrade through wind dispersal and gravitational sliding of unconsolidated scoria, whereas vent interiors are primarily affected by discrete rockfalls from oversteepened walls [48]. Finally, the overall edifice volume is modified by bulk subsidence driven by sedimentary compaction and thermal cooling contraction [48]. Such structural controls and erosion mechanisms can substantially modify the preserved cone volume and contribute to the observed variability in edifice morphology."

Comment 10:

Implications for hazard assessment. In section 3.6 the authors acknowledge that their study is retrospective and thus not applied during an active eruption. I agree with the author's discussion that this approach could be useful in some hazard assessments, but additional detail is needed. Specifically, how would the methods used here be used to support future hazard assessment? The approach presented does not appear to be feasible or relevant once an eruption has started. Therefore, would it be applied pre-eruption to assess where cones may form? Additional text is required in this section to make it clearer how the methods used in this study could be applied to future volcanic hazards.

Response:

We thank the reviewer for this excellent point. We completely agree that the true value of this methodology lies in pre-eruption spatial forecasting, however running these models from scratch during the rapid onset of an active fissure eruption is not feasible.To clarify how our approach could be applied prospectively, we have added ideas in a new paragraph in Section 3.6. The text now reads as follows **(Lines 575-585)**:

"The analytical approach that we propose is however retrospective, as the CSG thresholds that define the Permissive and Restrictive domains were obtained with the actual observations to maximize the fit. Prior to an eruption, such tuning is not possible. ***However, scenario-based forecasting can be achieved by testing possible values derived from historical analogues. For instance, applying the 0.23 MPa/km threshold from Lakagígar could serve to generate probabilistic hazard maps for future dykes fed by the Grímsvötn system. Alternatively, applying the 0.23 to 0.46 MPa/km threshold range observed across both eruptions could define Permissive and Restrictive envelopes for the broader EVZ. These ideas of approaching the hazard assessment are, however, speculative and require validation from other cases worldwide****."*

Comment 11:

Line numbers indicated in the responses always refer to the marked up document "Main_Revised_MarkedUp.pdf"

*"**Regional analysis** was based on the 2m × 2m ÍslandsDEM v.1.0, accessed via the …"* Specify.

We agree that the term "regional analysis" is vague. We have revised the sentence and completed it.

The sentence now reads (**Lines 615-617**):
"Regional-scale mapping of the eruptive cones and extraction of along-fissure elevation profiles were based on the 2m × 2m ÍslandsDEM v.1.0, accessed via the National Land Survey of Iceland (LMI)."

Comment 12:

"The original ArcticDEM models were generated from sub-meter commercial stereo imagery (e.g., WorldView 1-3 and GeoEye-1) processed using SETSM photogrammetric software [58]."

Somewhere in this first paragraph could you add a sentence for when this dataset was collected. I realize that your study area is well before any of these DEMs were generated but this may be relevant to future researchers trying to reproduce your study or build on it.

Response:

We thank the reviewer for pointing this out. Based on the official ÍslandsDEM metadata, we have added a sentence at the end of this paragraph specifying the multitemporal data collection period. The text now reads (**Lines 622-626**):

"The original ArcticDEM models were generated from sub-meter commercial stereo imagery (e.g., WorldView 1-3 and GeoEye-1) processed using SETSM photogrammetric software [73]. The satellite imagery used to generate these digital elevation models is multitemporal, typically acquired from 2012 to the present, with the oldest data dating back to 2008."

Comment 13:

Line 482-485: On one of the figures or in a new supplemental one, could you please display the area that the drone data covers? This will help readers clearly understand the spatial coverage of this dataset.

Response:

We thank the reviewer for the suggestion. Accordingly, we have updated Supplementary Figure S2 to clearly display the extent of the drone-covered area (**Fig. S2A in the Supplementary information file**). The figure caption now reads:

"*Fig. S2: Detailed view of vent distribution across two topographic highs along Lakagígar: L1 (the SW edge of the eruptive fissure) and L2 (Mount Laki). (A) Hillshade digital elevation*

model (DEM) of the Lakagígar area, the 226 delineated cones ***and the drone-covered area are indicated.*** *(...)”*

Comment 14:

Lines 491-493: *“The images were processed in Agisoft Metashape (v. 2.2) using Structure-from-Motion (SfM), producing a DEM and orthomosaic with a spatial resolution of ~20 cm/px.”*

Please report the vertical and horizontal accuracy of this dataset.

Response:

We thank the reviewer for this necessary correction. Based on our Post-Processing Kinematics (PPK) reports automatically generated on WingtraHub software, we have updated the manuscript to include the exact accuracy values.

The revised text now reads (**Lines 643-644**)

“The PPK geotagging of the dataset was done using WingtraHub v2.19.0 and achieved a mean absolute accuracy of 2 cm horizontally and 3 cm vertically.”

Comment 15: *In multiple locations within Section 4 the authors refer to the use of python libraries. This is useful but requires additional information to be relevant. Specifically, please state the analysis that these libraries were used to complete. Each library contains numerous processes and options, and simply stating the library used without context is unhelpful.*

Response:

We fully agree with the reviewer. We have revised the Section 4 of the manuscript and have stated the role of each library for our work. We also included the proper academic citations for all libraries used.

Specifically, in the Methods:

-Subsection “Pre-eruptive topography reconstruction” - Lines 701-703:
“To approximate the pre-eruptive topography, we processed these profiles in Python using the SciPy library [73] to smooth the topographic data and apply a spatial filtering (see Code Availability)”.

-Subsection “Topographic stress models” - Lines 735-739:
“We evaluated the magnitude and direction of the maximum Syy gradient, considering that magma flow is inhibited in the direction of increasing confining stress. The entire stress field and spatial gradients were computed through array operations using the NumPy library [76] in Python (see Code Availability).”

-Subsection "Statistical analysis" - Lines 742-748

"We implemented quantitative statistical workflows on our data using Python (see Code Availability). We used Pandas to manipulate the dataset [77], Scikit-learn for machine learning workflows [78], including the K-means clustering and the Receiver Operating Characteristic (ROC) analyses, SciPy for the peak-detection algorithms [73]; and Matplotlib [79] and Seaborn [80] for data visualization."
