# Supplementary material for: Topographic stress controls fissure segmentation and eruptive cone formation during the Laki and Eldgjá eruptions
Source: Commun Earth Environ. 2026 Sep 2;7(1):719. doi: 10.1038/s43247-026-03980-4 (PMC13538144; doi:10.1038/s43247-026-03980-4)
Supplement: Supplementary file 2 [file 43247_2026_3980_MOESM2_ESM.pdf]

Supplementary Information

This text provides extended methodological validation and statistical analyses supporting the findings in the main text. It includes (1) a comparative validation of our manual mapping approach against an automated detection algorithm (Netvolc, [1]) to justify the chosen mapping methods (Fig. S1); (2) detailed views within the Lakagígar eruptive fissure, detailing localised cone-slope interactions and fissure segmentations (Fig. S2); (3) detail on the statistical analyses used to select the Combined Stress Gradient (CSG) as the primary predictive metric (Figs. S3–S6); (4) depth sensitivity analyses demonstrating that our predictive models are valid across different target depths for sampling the stress metrics along the fissures (Figs. S7-S8); and (5) a continuous binning analysis approach applied to the Eldgjá fissure which includes the non-eruptive gaps as part of the statistical analysis presented in the main manuscript (Figs. S9-S11).

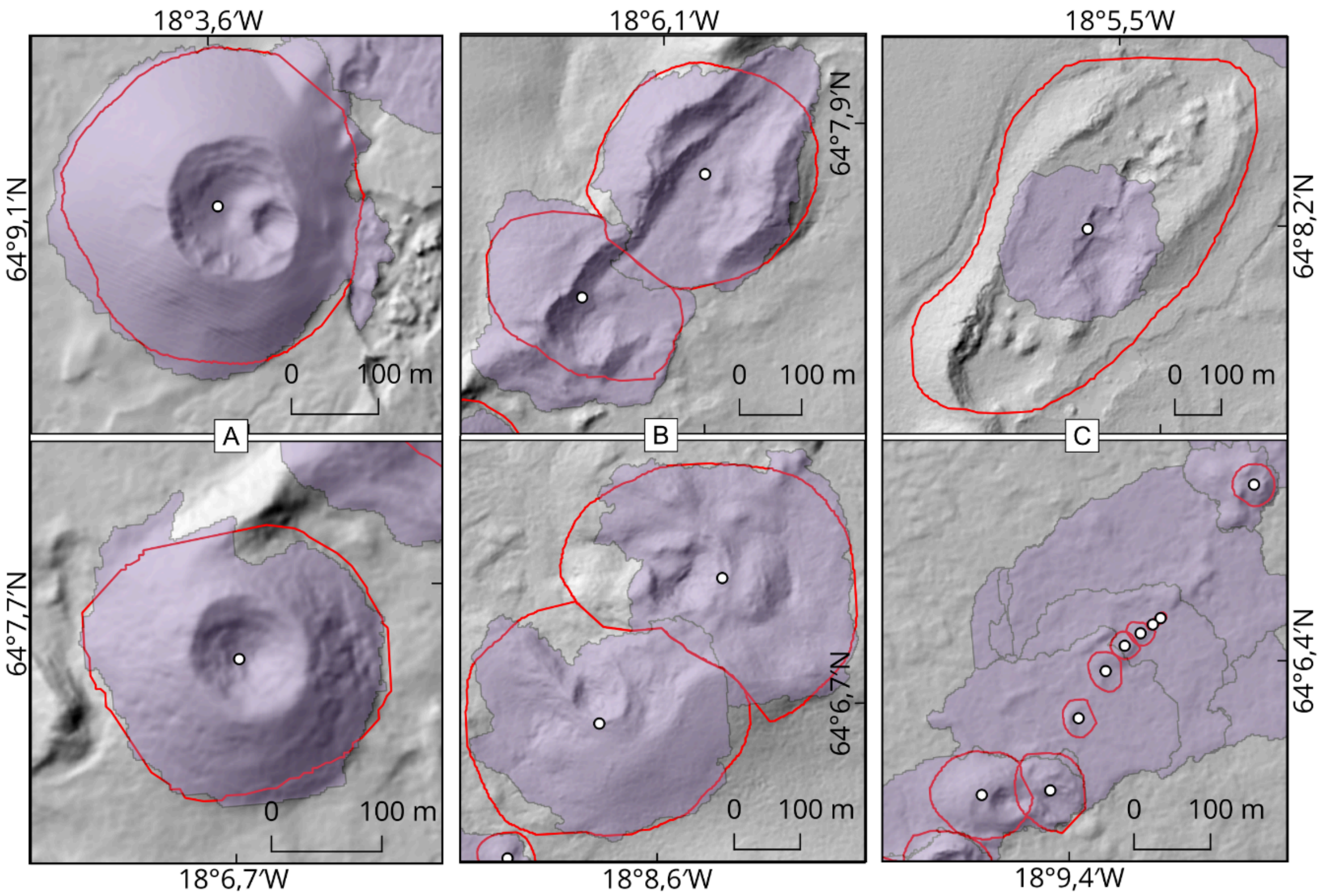

Fig. S1: Comparison between manual mapping (red line) and automated detection (purple polygons) using the NETVOLC algorithm[1] at Lakagígar, performed on a subset of 40 cones northeast of Mount Laki. Automated mapping uses an input window size of 200 px × 200 px (400 m × 400 m). (A) Example of successful automated detection on two isolated, conical landforms (representative of 3 cones in the complete dataset). (B) Example of cones mapped with moderate precision by the algorithm (representative of 5 cones in the complete dataset). (C) Example of very poor automated mapping, as the algorithm significantly under- or overestimates the basal limits (representative of the remaining 32 cones, or 80% of the tested subset). Because automated algorithms struggle with the complex, overlapping vent architecture characteristic of these fissure systems, manual digitisation based on Digital Elevation Model (DEM)-derived slope breaks was chosen for this study.

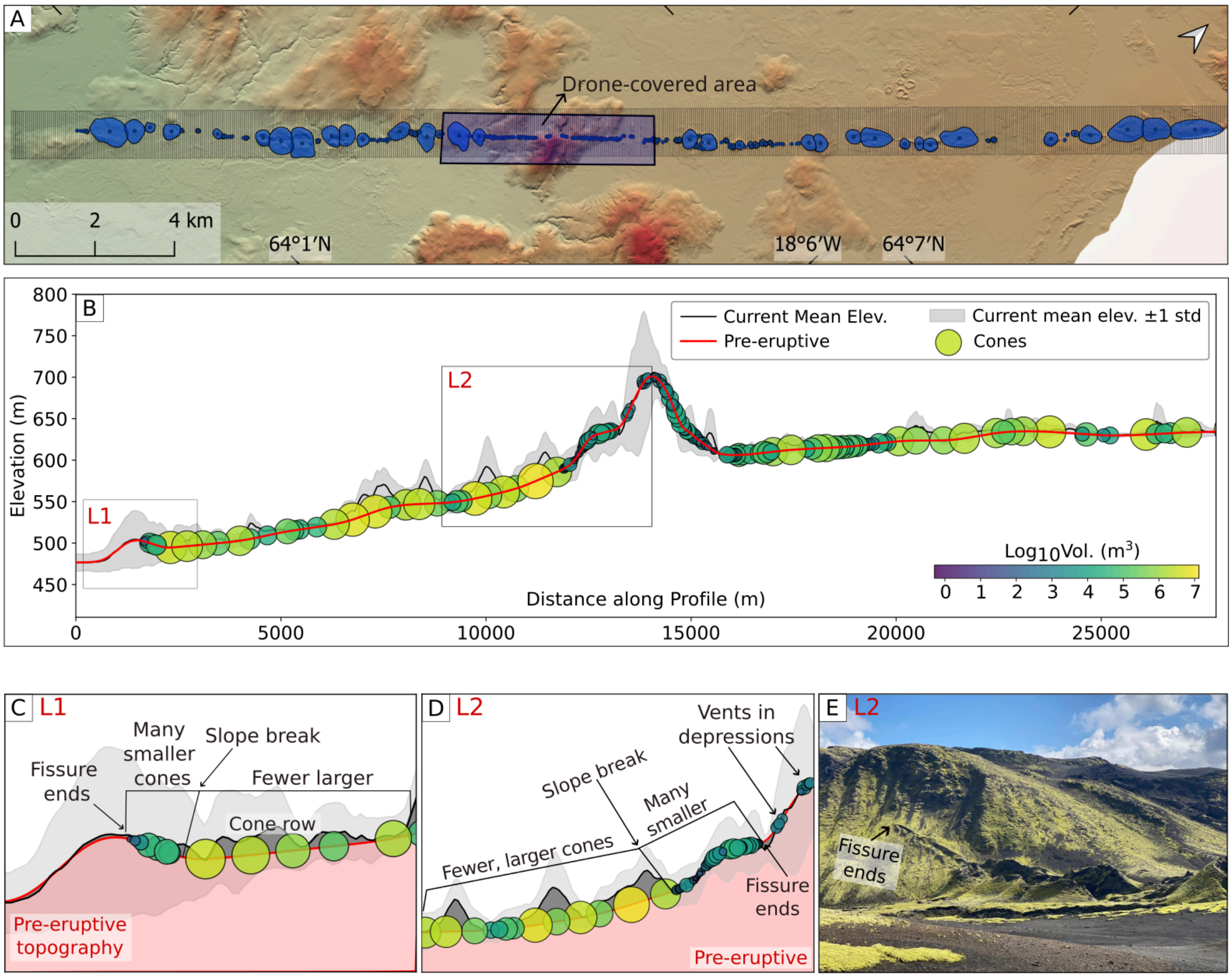

Fig. S2: Detailed view of vent distribution across two topographic highs along Lakagígar: L1 (the SW edge of the eruptive fissure) and L2 (Mount Laki). (A) Hillshade digital elevation model (DEM) of the Lakagígar area, the 226 delineated cones and the drone-covered area are indicated. (B) Pre-eruptive topography overlaid with cone centroids, where warmer colours indicate larger volumes. (C, D) Detail on cone volume clustering across the slope breaks of L1 (C) and L2 (D), defining zones of "Fewer, larger cones" versus "Many, smaller cones". In L2, vents disappear on the ascending slopes, reappear within small local depressions, and then resume on the opposite side of the topographic high. Because our pre-eruptive elevation profiles consider a 1200-m-wide elevation average, these localised depressions within L2 are smoothed out in the broader regional analysis. (E) Field photo showing cones becoming smaller as they climb up the SW slope of L2, until totally disappearing.

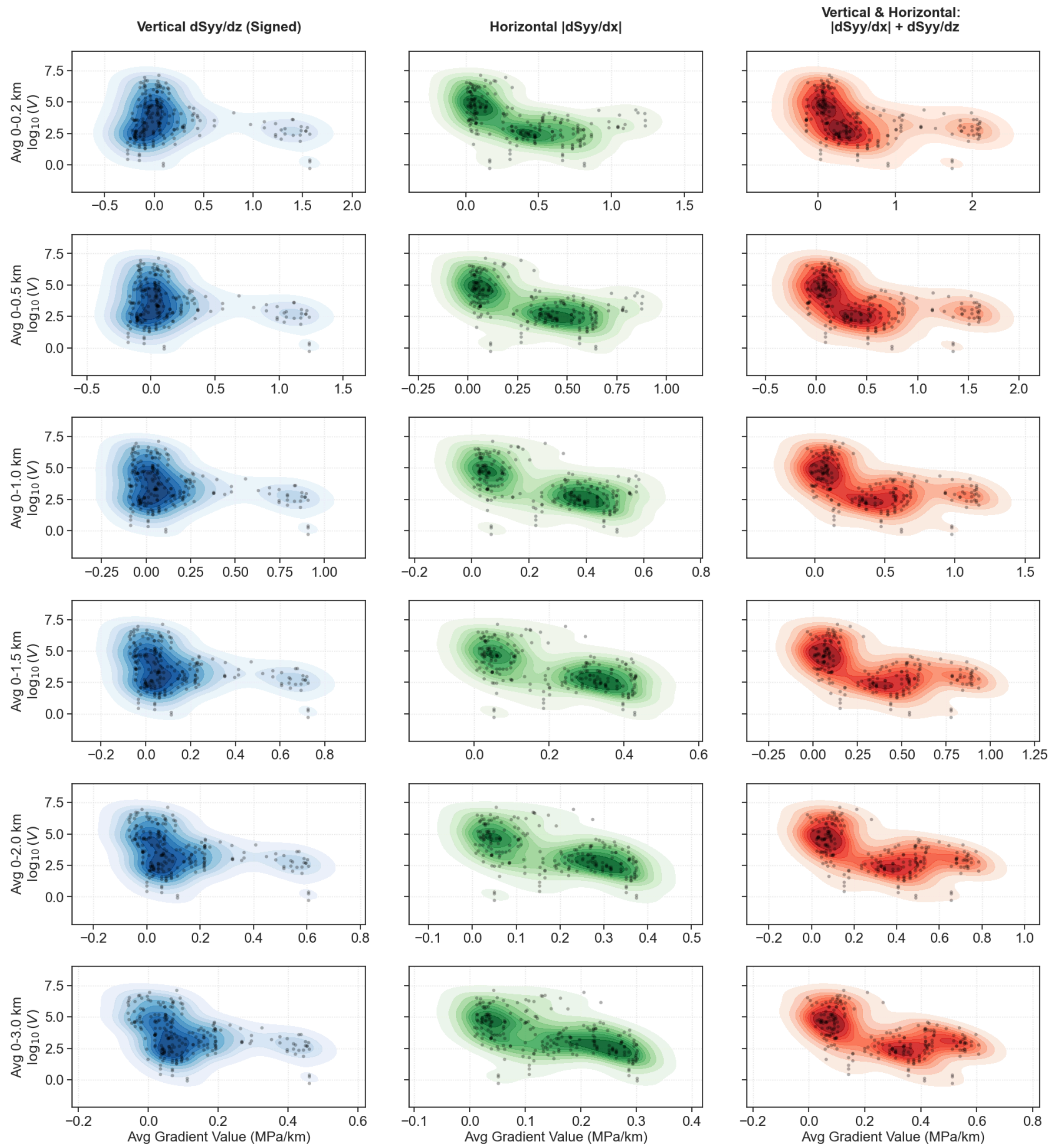

Fig. S3: Kernel Density Estimate (KDE) plots for Lakagígar evaluating the clustering capacity of logarithmic cone volumes ($\log_{10}$ V) against different stress metrics across various target depths (from 0–0.2 km to 0–3.0 km). The panels compare the vertical stress gradient (dS_{yy}/dz, in blue), the absolute horizontal stress gradient ($|dS_{yy}/dx|$, in green), and the Combined Stress Gradient (CSG, in red). The CSG provides the clearest segregation between small and large cone populations.

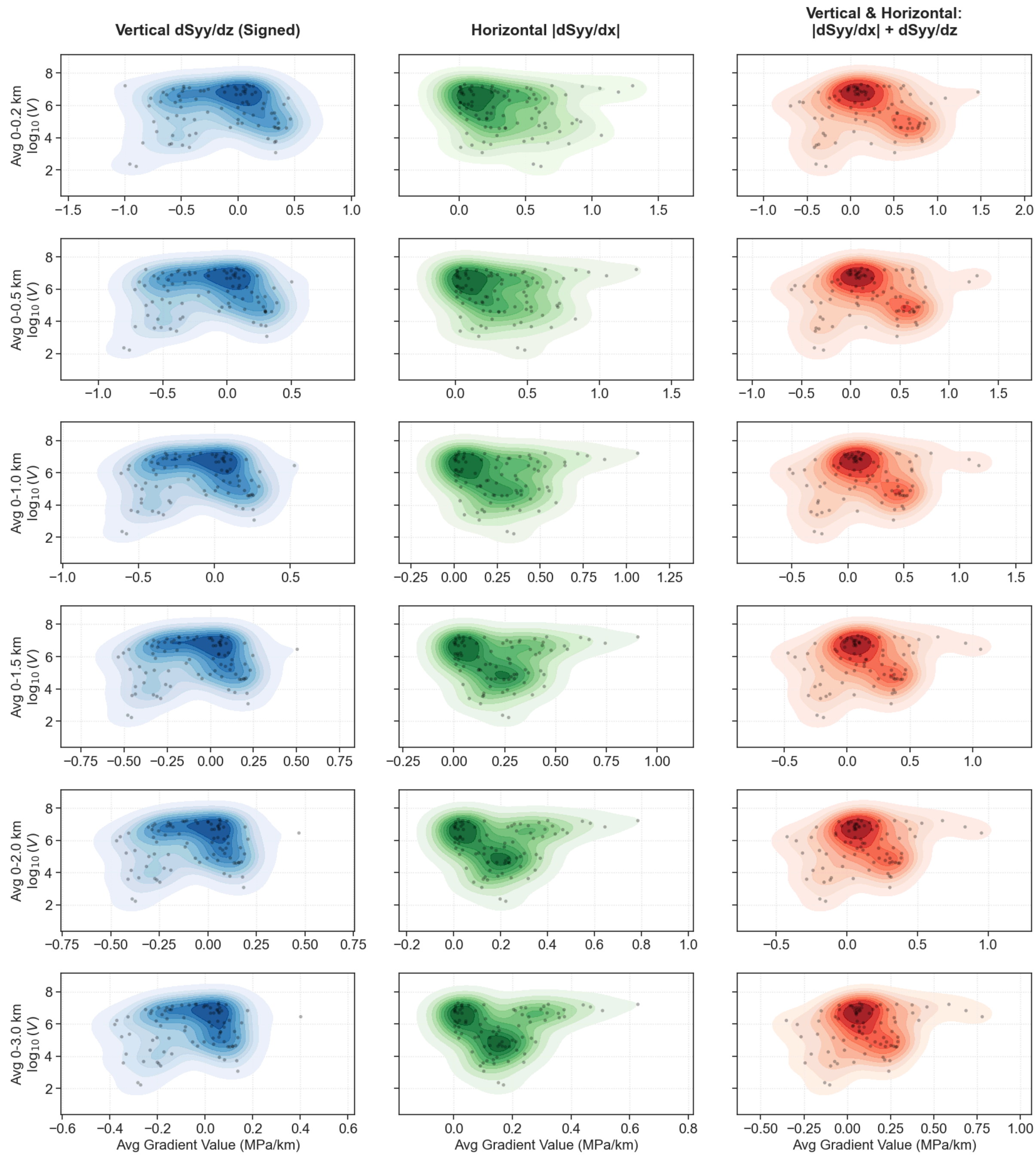

Fig. S4: Kernel Density Estimate (KDE) plots for Eldgjá evaluating the clustering capacity of logarithmic cone volumes ($\log_{10}$ V) against different stress metrics across various target depths (from 0–0.2 km to 0–3.0 km). The panels compare the vertical stress gradient (dS_{yy}/dz, in blue), the absolute horizontal stress gradient ($|dS_{yy}/dx|$, in green), and the Combined Stress Gradient (CSG, in red). The CSG provides the clearest segregation between small and large cone populations.

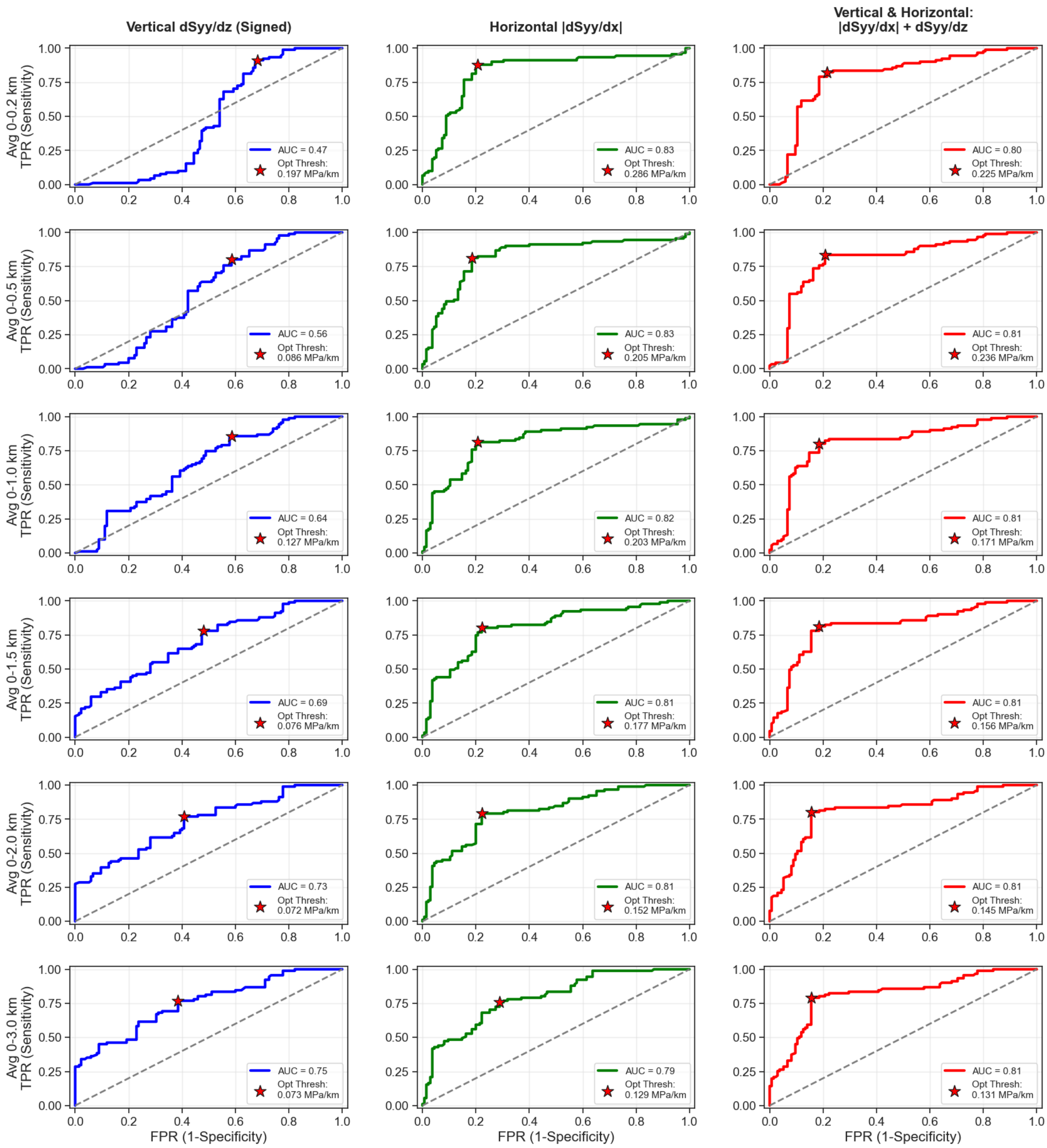

Fig. S5: Receiver Operating Characteristic (ROC) curves for Lakagígar evaluating the predictive performance of different stress metrics across various target depths (from 0–0.2 km to 0–3.0 km). The panels compare the vertical stress gradient (dS_{yy}/dz, in blue), the absolute horizontal stress gradient ($|dS_{yy}/dx|$, in green), and the Combined Stress Gradient (CSG, in red) in classifying large versus small cone populations. TPR stands for True Positive Rate (Sensitivity), and FPR stands for False Positive Rate (1 - Specificity). Red stars indicate the optimal threshold value for each metric (maximum Youden's index). The CSG presents a high Area Under the Curve (AUC) value across all depths (~0.83), providing a strong discrimination capacity between Permissive and Restrictive domains.

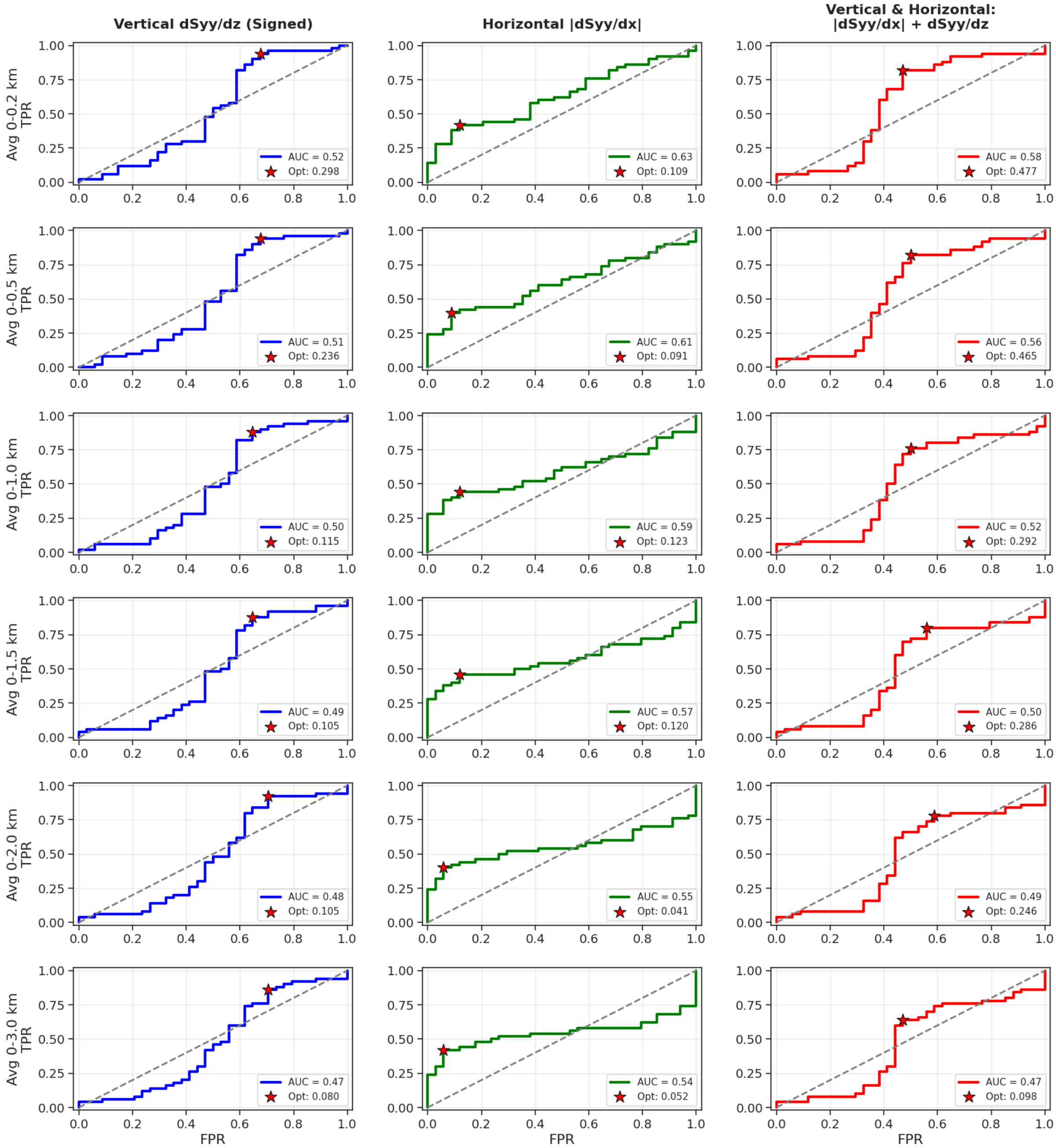

Fig. S6: Receiver Operating Characteristic (ROC) curves for Eldgjá evaluating the predictive performance of different stress metrics across various target depths (from 0–0.2 km to 0–3.0 km). The panels compare the vertical stress gradient (dS_{yy}/dz, in blue), the absolute horizontal stress gradient ($|dS_{yy}/dx|$, in green) and the Combined Stress Gradient (CSG, in red) in classifying large versus small cone populations. TPR stands for True Positive Rate (Sensitivity), and FPR stands for False Positive Rate (1 - Specificity). Red stars indicate the optimal threshold value for each metric (maximum Youden's index). The overall Area Under the Curve (AUC) values are lower for Eldgjá (~ 0.5 – 0.6) compared to Lakagígar, attributed to the smaller number of samples, as the dominant mechanism in Eldgjá due to stress is the fissure segmentation (gaps).

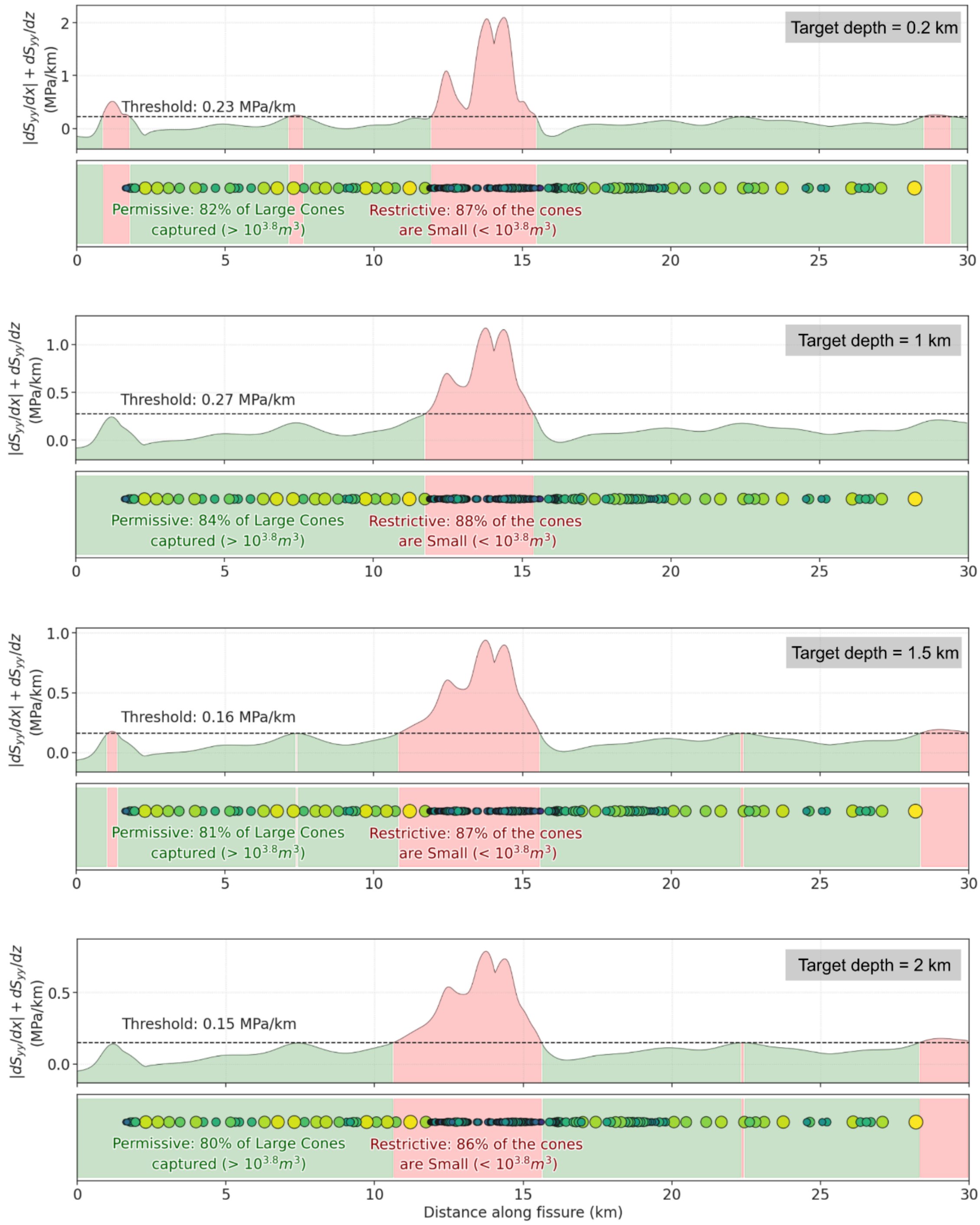

Fig. S7: Depth sensitivity analysis of the predictive model for Lakagígar, evaluating the stability of the Combined Stress Gradient across four target depths (0.2 km, 1 km, 1.5 km and 2 km). The figures mimic Fig. 4 (main text). For each depth, the upper panel shows the CSG ($|dS_{yy}/dx| + dS_{yy}/dz$) variation along the fissure, with the optimal threshold (from the ROC analysis) indicated by a dashed line. The lower panel displays the resulting Permissive and Restrictive domains against the spatial distribution of the observed cone volumes. The Permissive domains (green), successfully capture over 80% of the large-volume cones in all cases, and Restrictive domains (red) present a vast majority of small cones. The consistent spatial distribution of these domains and the high capture rates demonstrate that the model's validity is robust and independent of the specific depth used to average the CSG.

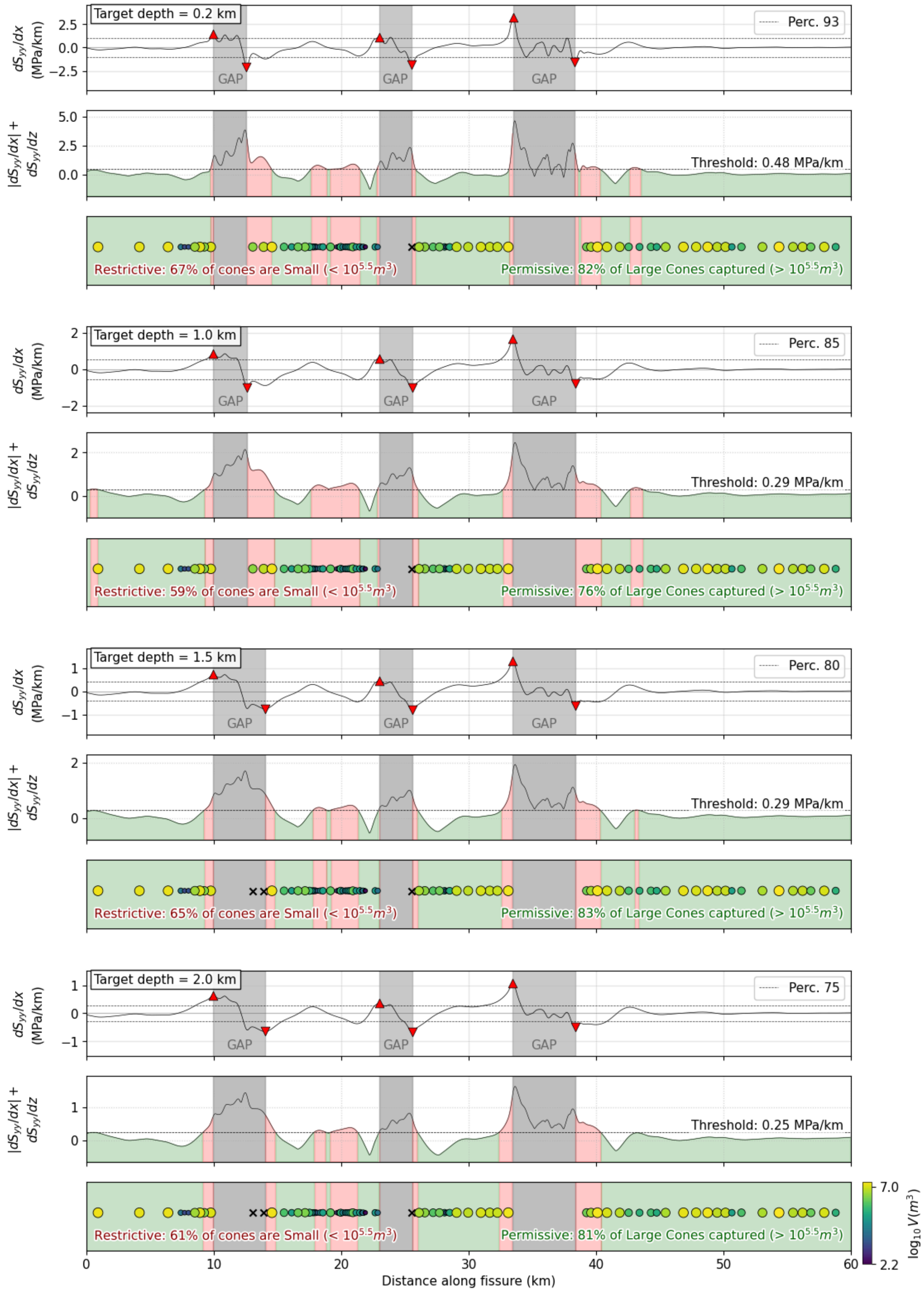

Fig. S8: Depth sensitivity analysis of the predictive model for Eldgjá across four target depths (0.2, 1, 1.5, and 2 km), mimicking Fig. 5 (main text). Upper and middle panels show the horizontal stress gradient (dS_{yy}/dx) and CSG variations along the fissure, highlighting predicted structural gaps (grey shading) and optimal thresholds (dashed lines, corresponding to the 93rd, 85th, 80th and 75th percentiles respectively). The lower panel plots Permissive (green) and Restrictive (red) domains against observed cone volumes. Permissive domains consistently capture the majority of large cones, while Restrictive domains and gaps capture small or no cones (black crosses). These spatial distributions remain independent of the specific averaging depth.

Continuous spatial binning analysis for Eldgjá

In the cone volume statistical analysis for Eldgjá presented in the main text (see section 'Eldgjá: cone volume distribution and fissure-gap prediction'), the predictive performance was low compared to Lakagígar (AUC = 0.56). This lower performance is related to the presence of kilometre-scale gaps lacking eruptive cones, where very high stress gradients completely arrest the dyke at depth. Because our initial calculation considered only existing cones, it did not account for this complete eruption inhibition (zero-volume areas). This reduced the number of small-volume data points, impacting the AUC.

Methods

To address this, we present a complementary approach where eruptive gaps are considered in the statistical evaluation. We implemented a spatial binning approach where we evaluated the entire Eldgjá fissure using a sliding window. The division between small and large cones is preserved as original, defined from the K-Means clustering algorithm exclusively applied to the observed individual cones, with a threshold set at a volume of $10^{5.5}$ m^3. For each bin, we calculate the mean volume of all cone centroids falling within the window, assigning a volume of 0 m^3 to bins covering eruptive gaps, and extract the corresponding mean combined stress gradient (CSG) for each bin. Finally, we apply a ROC curve analysis to evaluate the model's performance now that we are considering the gaps in our analysis, to predict whether a bin would behave as a *Permissive* (mean volume > $10^{5.5}$ m^3) or *Restrictive* domain based on the CSG.

Results

We present the results of this analysis using three different window sizes (1, 1.5 and 2 km), maintaining a proportional step size of 50% of the window size in all configurations (0.5, 0.75 and 1 km, respectively). The overall performance of the Combined Stress Gradient (CSG) model, represented by the AUC, is consistently improved with respect to the discrete statistical model presented in the main text.

First configuration, 1 km window, 0.5 km step (Fig. S9). The scatter plot (Fig. S9C) displays a high density of data points due to the small step size and high spatial resolution (Fig. S9E). This configuration yields an AUC of 0.67 with an optimal CSG threshold of 0.42 MPa/km. It successfully captures 78% of the large volume bins in the Permissive domains and 59% of gaps/small bins in Restrictive domains. The low accuracy in Restrictive domains results from the window overlapping effect: bins centred on the boundaries of the Restrictive domains overlap with large cones of the adjacent permissive regions and artificially increase the bin's mean volume, increasing the number of false negatives (Fig. S9C, E).

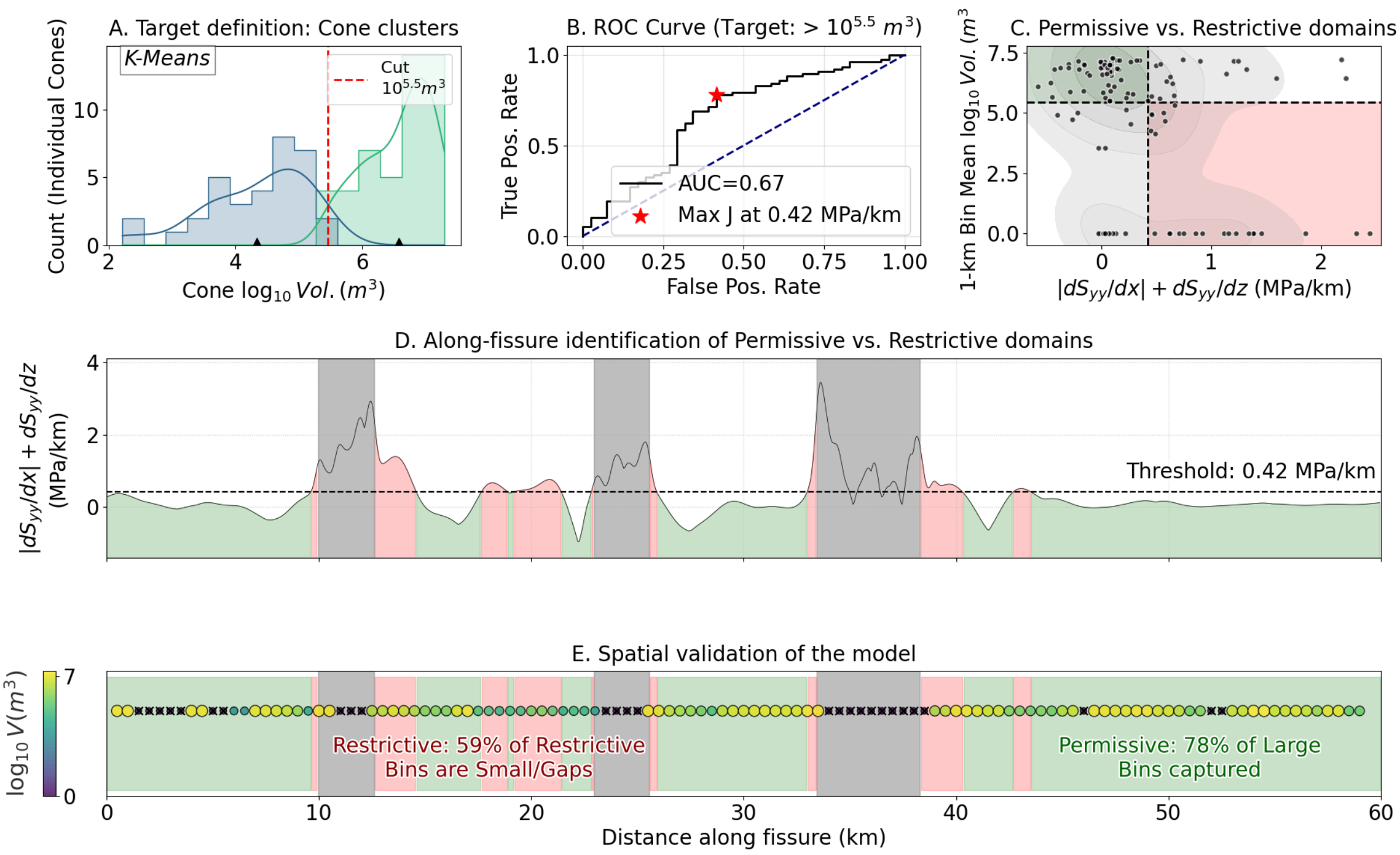

Fig. S9. Continuous binning analysis of the Eldgjá fissure using a 1 km sliding window and 0.5 km step size. (A) K-Means clustering defining the small-large volume threshold at $10^{5.5}$ m³. (B) ROC curve analysis showing an AUC of 0.67 based on the Combined Stress Gradient (CSG), with the red star indicating the maximum Youden's J index (Max J). (C) Scatter plot of mean bin volume vs. CSG, dividing data into Permissive (green) and Restrictive (red) domains using the optimal CSG threshold at 0.42 MPa/km. (D) Along-fissure CSG profile and optimal threshold applied spatially. (E) Spatial validation of the model, capturing 78% of large bins in Permissive domains and 59% of small/gap bins captured in Restrictive domains.

Second configuration, 1.5 km window, 0.75 km step (Fig. S10). This configuration yields the best performance of the three configurations (AUC=0.72). The spatial resolution is increased, which minimises the local noise, but preserves the magnitude of the stress peaks. The optimal CSG threshold is kept almost constant at 0.40 MPa/km. At this resolution, the model achieves a capture rate of 81% for Permissive domains and 58% for Restrictive domains (Fig. S10E).

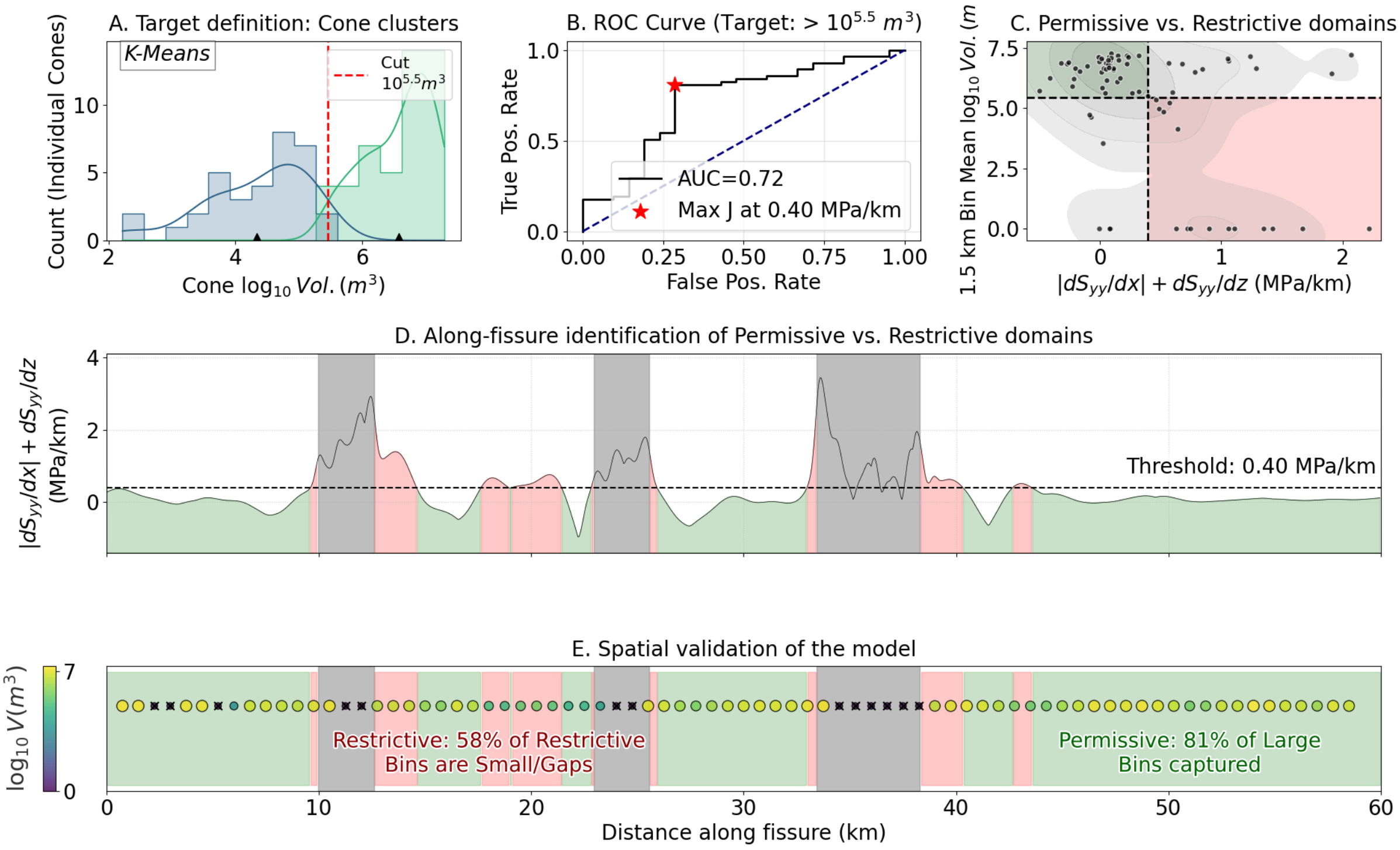

Fig. S10. Continuous binning analysis of the Eldgjá fissure using a 1.5 km sliding window and 0.75 km step size. (A) K-Means clustering defining the small-large volume threshold at $10^{5.5}$ m³. (B) ROC curve analysis showing an AUC of 0.72 based on the Combined Stress Gradient (CSG), with the red star indicating the maximum Youden's J index (Max J). (C) Scatter plot of mean bin volume vs. CSG, dividing data into Permissive (green) and Restrictive (red) domains using the optimal CSG threshold at 0.40 MPa/km. (D) Along-fissure CSG profile and optimal threshold applied spatially. (E) Spatial validation of the model, capturing 81% of large bins in Permissive domains and 58% of small/gap bins captured in Restrictive domains.

Third configuration, 2 km window, 1 km step (Fig. S11). As the window size increases to 2 km, the boundary overlapping effect becomes stronger, reducing the model's overall performance to an AUC of 0.65. The capture rate of large cones in Permissive domains drops to 69% and only 39% of bins falling in Restrictive domains are small/gaps. At this scale, if we center the window over a gap, it is so wide that it also includes the large cones sitting just outside of it, while the extreme CSG values inside the gaps are smoothed by the stress magnitudes of the surrounding areas. This causes the CSG threshold to drop significantly to 0.28 MPa/km.

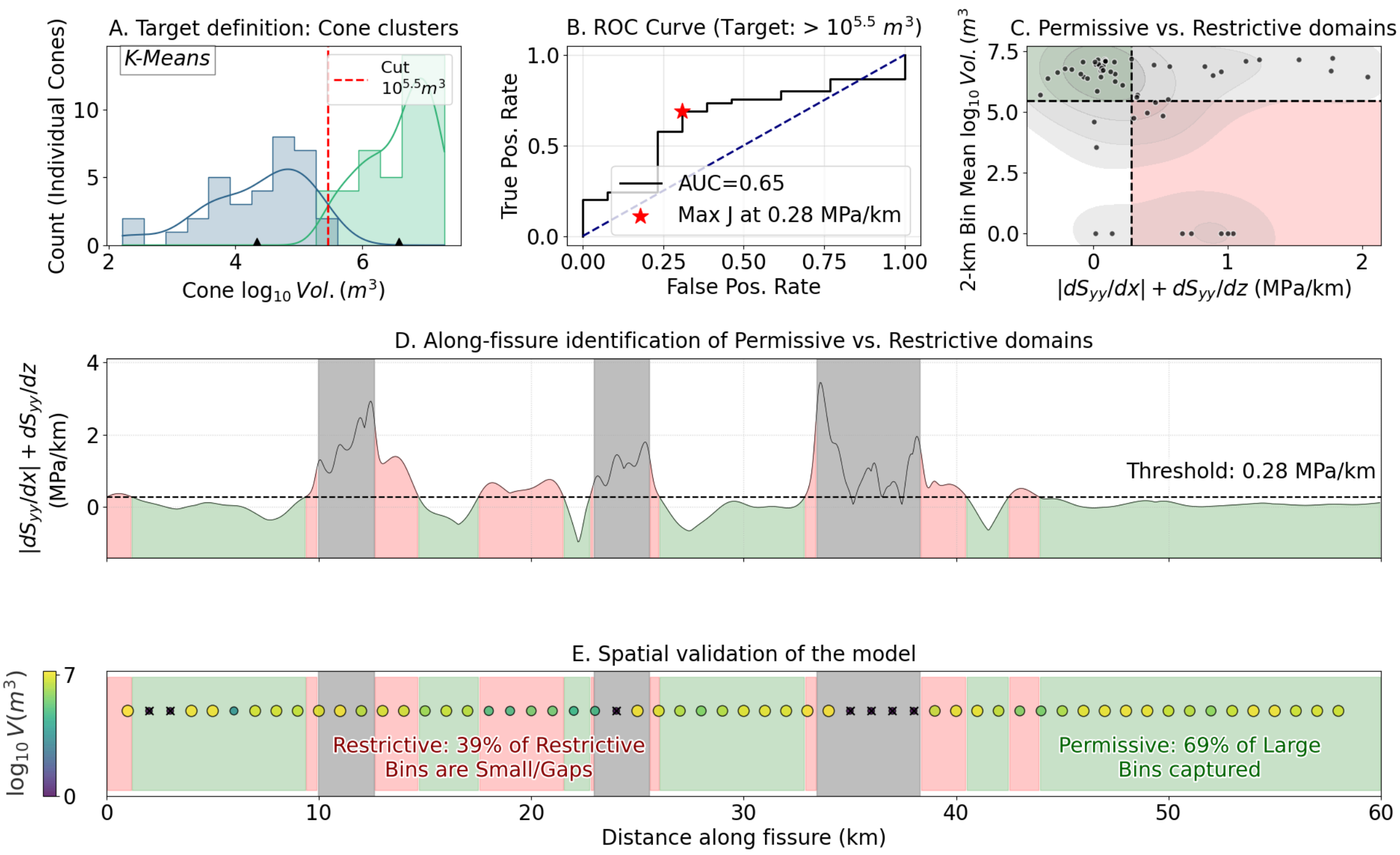

Fig. S11. Continuous binning analysis of the Eldgjá fissure using a 2 km sliding window and 1 km step size. (A) K-Means clustering defining the small-large volume threshold at $10^{5.5}$ m³. (B) ROC curve analysis showing an AUC of 0.65 based on the Combined Stress Gradient (CSG), with the red star indicating the maximum Youden's J index (Max J). (C) Scatter plot of mean bin volume vs. CSG, dividing data into Permissive (green) and Restrictive (red) domains using the optimal CSG threshold at 0.28 MPa/km. (D) Along-fissure CSG profile and optimal threshold applied spatially. (E) Spatial validation of the model, capturing 69% of large bins in Permissive domains and 39% of small/gap bins captured in Restrictive domains.

Overall, the AUC is consistently improved, while the CSG threshold remains stable (~0.30-0.42 MPa/km), provided the spatial resolution is carefully balanced according to the system dimensions. Here we show that the window size choice is a trade-off: while larger windows reduce the noise, excessively large windows make the boundary effect dominate and hide the high topographic stress values related to the gaps. Therefore, scaling the analytical window to the physical dimensions of the geological features is essential for maintaining a statistical validity of the model.

<u>References</u>

1. Euillades, L. D., Grosse, P. & Euillades, P. A. NETVOLC: An algorithm for automatic delimitation of volcano edifice boundaries using DEMs. Computers & Geosciences 56, 151-160 (2013).
